# Patterns of granulocyte colony–stimulating factor prophylaxis in patients with cancer receiving myelosuppressive chemotherapy

**DOI:** 10.1007/s00520-020-05295-2

**Published:** 2020-01-10

**Authors:** Prasad L. Gawade, Shuling Li, David Henry, Nancy Smith, Rajesh Belani, Michael A. Kelsh, Brian D. Bradbury

**Affiliations:** 1 Center for Observational Research, Amgen Inc., One Amgen Center Drive, Thousand Oaks, CA 91320 USA; 2 Chronic Diseases Research Group, Hennepin Healthcare Research Institute, Minneapolis, MN USA; 3 Department of Medicine, University of Pennsylvania, Philadelphia, PA USA; 4 DOCS Global, Waltham, MA USA; 5 US Medical Affairs, Amgen Inc., Thousand Oaks, CA USA

**Keywords:** Febrile neutropenia, Filgrastim, Granulocyte colony–stimulating factor, Pegfilgrastim, Prophylaxis, On-body injector

## Abstract

**Purpose:**

To evaluate patterns of primary prophylactic (PP) granulocyte colony–stimulating factor (G-CSF) use following chemotherapy by cancer type and febrile neutropenia (FN) risk.

**Methods:**

Using a commercial administrative database, we identified adult patients diagnosed with breast, colorectal, lung, ovarian cancer, or non-Hodgkin lymphoma (NHL) who initiated chemotherapy with high risk (HR) or intermediate risk (IR) for FN between January 1, 2013, and August 31, 2017. We describe use of PP-G-CSF, proportion completing all their cycles with pegfilgrastim, timing of pegfilgrastim, and duration of short-acting G-CSF.

**Results:**

Among 22,868 patients (breast 11,513; colorectal 3765; lung 4273; ovarian 1287; and NHL 2030), 36.8% received HR and 63.2% received IR (64.4% of whom had ≥ 1 risk factor [RF] for FN). Proportions of patients receiving PP-G-CSF in the first cycle were 76.1%, 28.2%, and 26.4% among patients receiving HR, IR, and IR plus ≥ 1 RF, respectively. Among breast cancer patients receiving HR regimens and initiating PP-pegfilgrastim, 60.4% (95% confidence interval [CI] 57.2–63.6%) initiating via on-body injector (OBI) and 51.9% (95% CI 48.0–55.8%) initiating via prefilled syringe (PFS) completed all their cycles with OBI and PFS, respectively. Among all cycles with PP-PFS, 8.5% received PFS on the same day as chemotherapy completion. Mean administrations/cycle were 3.2 (standard deviation [SD] 2.3) for filgrastim, 3.0 (SD 1.6) for filgrastim-sndz, and 4.3 (SD 2.5) for tbo-filgrastim.

**Conclusions:**

There is under- and mistimed use of PP-G-CSF among patients at HR for FN. Novel pegfilgrastim delivery devices could help breast cancer patients at HR for FN complete all their cycles with timely prophylaxis.

**Electronic supplementary material:**

The online version of this article (10.1007/s00520-020-05295-2) contains supplementary material, which is available to authorized users.

## Introduction

Scientific discovery is leading to rapid advances in our understanding of cancer and is delivering new targeted medicines [1–3], leading to substantial clinical benefit. Despite these advances, most patients diagnosed with cancer today are still treated with chemotherapy [4] and are thus at risk of experiencing chemotherapy-related adverse effects such as fatigue, infusion reactions, cognitive dysfunction, cardiovascular or gastrointestinal toxicity, and febrile neutropenia (FN) [5, 6]. Patients diagnosed with solid tumors who develop FN are frequently hospitalized for multiple days at a time [7] often resulting in delays or modifications to scheduled chemotherapy sessions [8], and in some instances, more severe complications including death [9]. The National Comprehensive Care Network (NCCN®) clinical practice guidelines [10] recommend prophylactic use of granulocyte colony–stimulating factor (G-CSF) to promote the growth and differentiation of neutrophils for patients receiving chemotherapy regimens associated with a high risk of developing FN (> 20%) or regimens with intermediate risk of FN (10–20%) and ≥ 1 patient-level risk factor.

There have been important developments in recent years regarding FN management, including greater focus placed on high-quality, low-cost cancer care through efforts such as the Oncology Care Model (OCM) by the Centers for Medicare and Medicaid Services (CMS) [11], the entrance of biosimilars of filgrastim [12–14] and pegfilgrastim [15, 16] in the USA, and the introduction of new, patient-centric drug delivery devices [17]. The effects that these changes have had on prescribing patterns and persistence with chemotherapy regimens are largely unknown but are important to establish for future requisite investigations into the clinical consequences of such therapeutic decisions.

To that end, we used data from a nationally representative population of patients with commercial and Medicare Advantage insurance to evaluate the patterns of G-CSF prophylaxis in patients diagnosed with various types of cancer and receiving chemotherapy regimens with high/intermediate FN risk.

## Methods

### Study design and data source

We used a retrospective cohort study design. The data used in this study came from the Optum™ Clinformatics™ Data Mart (Optum Insight, Eden Prairie, MN) which has been described previously [18]. Briefly, the Optum™ Clinformatics™ Data Mart includes de-identified eligibility, pharmacy, laboratory, medical, and standard pricing data for approximately 15 million people enrolled annually in commercial and Medicare advantage plans by United Health Group and is fully compliant with the Health Insurance Portability and Accountability Act of 1996 [19]. Approximately 20% of Medicare advantage patients in the USA are represented in this database. The population is geographically diverse and spans all 50 states. We used the Date of Death (DOD) data series of the Optum™ Clinformatics™ Data Mart that includes dates of death obtained from the Social Security Administration death master file.

### Study population

We identified patients ≥ 18 years of age diagnosed with breast, colorectal, lung, ovarian cancer, or non-Hodgkin lymphoma (NHL) who had initiated myelosuppressive chemotherapy regimens with high/intermediate risk for FN as defined in the 2017 v2. NCCN® clinical practice guidelines [20] (Online Resource 1) from January 1, 2013, to August 31, 2017. Initiation of myelosuppressive chemotherapy was defined as first observed myelosuppressive chemotherapy treatment during the study period without any evidence of myelosuppressive chemotherapy during the preceding 365 days. The date of myelosuppressive chemotherapy initiation was assigned as the index date. All patients were required to have ≥ 365 days of continuous enrollment prior to the index date (i.e., baseline period) to assess patient characteristics and comorbidities (Online Resource 2). The study follow-up period started on the index date and ended on the earliest date of last day of the last cycle of chemotherapy course, last day of cycle 8 of chemotherapy course, occurrence of FN-related hospitalization (≥ 1 inpatient diagnosis claim in any position), bone marrow or stem cell transplant or radiation therapy (≥ 1 inpatient or outpatient diagnosis/procedure claim in any position) (Online Resource 3), death, disenrollment from commercial plan, or December 31, 2017.

We categorized patients by the cancer diagnosis most proximal to the index date of the myelosuppressive chemotherapy (within 365 days preceding and 30 days following the index date) based on the presence of 1 inpatient diagnosis or 2 outpatient diagnoses (separated by ≥ 7 days) from the International Classification of Diseases 9th/10th Revision Clinical Modification (ICD-9-CM/ICD-10-CM) codes (Online Resource 4). Cancer diagnosis was identified using modification of a commonly used algorithm in administrative claims research requiring at least 1 inpatient or at least 2 outpatient diagnoses claims [21]. Because this algorithm has reported high specificity but moderate sensitivity, we required the 2 outpatient diagnoses claims to be ≥ 7 days apart and allowed for a 30-day window beyond the index date to improve its sensitivity. Calendar year cohorts of patients with 5 distinct cancer types were identified: breast, colorectal, lung, ovarian, and NHL. Patients were required to have survived the first 6 days after the index date to ensure enough time to identify the chemotherapy regimen and day of chemotherapy completion in the first chemotherapy cycle. Claims of appropriate injectable chemotherapeutic agents were identified at specified intervals for each chemotherapy regimen and its variations as defined in the source publication listed in NCCN® guidelines (Online Resource 5) [20]. Dose of chemotherapeutic agents was unavailable in the claims database. We excluded patients with ≥ 1 type of non-myeloid cancer diagnosis, and patients with evidence of bone marrow or stem cell transplant or radiation therapy during the baseline period.

### Covariate assessment

Age in years and sex were assessed at the index date. Comorbidities were defined based on the presence of ≥ 1 diagnosis claim in an inpatient setting or ≥ 2 diagnosis claims in an outpatient setting (separated by ≥ 30 days) during the baseline period. Neutropenia hospitalization was defined as an inpatient stay with a diagnosis of neutropenia (ICD-9 288.0X; ICD-10 D70.X) in any position. The modified Charlson comorbidity index (CCI) score, adapted for use with administrative claims data and including updated severity weights, was calculated [22, 23].

Patient-level FN risk factors that were assessed during baseline included age > 65 years, liver or renal dysfunction, human immunodeficiency virus/acquired immunodeficiency syndrome (HIV/AIDS) identified by NCCN® guidelines [10], and also cardiovascular disease (defined as myocardial infarction, heart failure, peripheral vascular disease, or stroke), chronic obstructive pulmonary disease, diabetes mellitus, and metastatic disease considered as FN risk factors by the authors (Online Resource 3).

### Outcome assessment

Prophylactic G-CSF was defined as the receipt of ≥ 1 administration of G-CSF from the chemotherapy initiation date up to 5 days following the last chemotherapy administration in the first cycle. Short-acting G-CSFs (sG-CSFs; filgrastim, filgrastim-sndz, and tbo-filgrastim) were identified using established Healthcare Common Procedure Coding System (HCPCS) and National Drug Code (NDC) numbers (Online Resource 6). Number of prophylactic sG-CSF administrations was defined as number of days with sG-CSF claims during the entire cycle; multiple claims on the same day were considered a single administration. NDC and Current Procedural Terminology (CPT) codes were used to distinguish between two modes of pegfilgrastim administration: prefilled syringe (PFS) and on-body injector (OBI) (Online Resource 7). The OBI is applied to the patient’s abdomen or arm on the same day as chemotherapy to automatically deliver pegfilgrastim approximately 27 h after application. Claims with HCPCS codes for pegfilgrastim alone were categorized as “pegfilgrastim, route unknown.” Timing for administration of prophylactic pegfilgrastim PFS or OBI was identified by a claim for pegfilgrastim PFS or OBI relative to day 0 (i.e., same day as chemotherapy completion) through day 5 after chemotherapy completion. Use of prophylactic G-CSF in the second and all subsequent cycles was defined similarly. For each eligible patient in the study cohort, the number of completed chemotherapy cycles (i.e., cycles identified within a course) up to 8 was identified.

### Statistical analysis

Descriptive statistics for continuous variables included mean and standard deviation (SD) and median and interquartile range (quartile 1 [Q1] and quartile 3 [Q3]), and categorical variables included count (*n*) and percentages (%) with 95% binomial confidence intervals (CIs). Standardized differences were calculated to compare age, sex, and comorbidities of patients receiving versus not receiving G-CSF. Results were stratified by calendar year, cancer type, and risk category of the chemotherapy regimen. Prophylactic G-CSF use was assessed by number and proportion across all cycles during the chemotherapy course. The mean (SD) number of prophylactic sG-CSF administrations per cycle was calculated using all eligible cycles that received ≥ 1 administration of prophylactic sG-CSF.

During the post-OBI approval period (i.e., after March 1, 2015), persistence of prophylactic pegfilgrastim was calculated for PFS and OBI following chemotherapy completion. Among patients who received prophylactic pegfilgrastim in the first cycle, persistence of prophylactic pegfilgrastim was calculated as proportion of patients completing 100% of their cycles (up to 8 cycles) with the same type of pegfilgrastim they used in first cycle.

## Results

The source population from which patients with the 5 cancer types were identified ranged from 13.3 million in 2013 to 16.4 million in 2017. A total of 22,868 cancer patients (11,513 breast; 4273 lung; 3765 colorectal; 2030 NHL; and 1287 ovarian) met the study inclusion criteria (Online Resource 8). Among these patients, 36.8% were receiving chemotherapy regimens with high FN risk and 63.2% were receiving chemotherapy regimens with intermediate FN risk (among these, 64.4% had ≥ 1 FN risk factor) (Online Resource 8). Chemotherapy regimens with high FN risk were most common in breast cancer patients (71.8%) compared with ≤ 6% for patients with other cancer types (Online Resource 8).

Overall and across cancer types among patients receiving chemotherapy with high FN risk, comorbidity profiles were similar between those who received versus those who did not receive prophylactic G-CSF (Table 1, Online Resource 8). Among all patients with intermediate FN risk (35.0% versus 20.2%) and intermediate FN risk with ≥ 1 risk factor (53.0% versus 33.6%), those not receiving prophylactic G-CSF were more likely to have a baseline metastatic diagnosis compared to patients receiving prophylactic G-CSF. The high likelihood of baseline metastasis for patients not receiving prophylactic G-CSF and receiving chemotherapy regimens with intermediate FN risk was observed only for breast cancer but was not evident among other cancer types (Table 1, Online Resource 8).

**Table 1:** Baseline demographics and comorbidities of eligible study population receiving chemotherapy with high/intermediate FN risk stratified by cancer type, FN risk category of the chemotherapy regimen, and receipt of primary prophylactic G-CSF

	Breast cancer			Colorectal cancer			Lung cancer			NHL			Ovarian cancer		
	Prophylactic G-CSF			Prophylactic G-CSF			Prophylactic G-CSF			Prophylactic G-CSF			Prophylactic G-CSF		
	No	Yes	Std diff	No	Yes	Std diff	No	Yes	Std diff	No	Yes	Std diff	No	Yes	Std diff
High risk	*N* = 8270			*N* = 0			*N* = 4			*N* = 114			*N* = 36		
Proportion, row %	23.6	76.4		–	–	–	75.0	25.0		23.7	76.3		86.1	13.9	
Age, years (SD)	59.2 (11.7)	58.8 (12.0)	− 0.03	–	–	–	75.3 (4.7)	76.0 (−)	–	55.9 (15.6)	60.3 (13.8)	0.30	66.3 (12.3)	72.0 (11.1)	0.49
Sex, female	100.0	100.0	0	–	–	–	66.7	100	− 2.0	18.5	37.9	0.44	100.0	100.0	0
Comorbidities															
CVD	4.6	4.9	0.01	–	–	–	0	0	–	25.9	17.2	− 0.21	22.6	20	− 0.06
CKD	3.7	3.2	− 0.03	–	–	–	0	0	–	11.1	9.2	− 0.06	6.5	20	0.41
COPD	1.7	1.7	0	–	–	–	33.3	0	− 1.0	3.7	3.4	− 0.01	0	40	1.16
Diabetes type 2	15.9	15.0	− 0.03	–	–	–	0	0	–	22.2	21.8	− 0.01	22.6	0	− 0.76
Liver disease	1.3	1.1	− 0.02	–	–	–	0	0	–	3.7	6.9	0.14	3.2	0	− 0.26
Metastasis	9.5	12.1	0.08	–	–	–	0	0	–	7.4	4.6	− 0.12	61.3	40	− 0.44
NRH	0.2	0.1	− 0.01	–	–	–	0	0	–	7.4	2.3	− 0.24	0	0	–
Charlson comorbidity index	4.6 (3.3)	5.0 (3.3)	0.12	–	–	–	7.0 (4.4)	2.0 (−)	–	4.2 (2.9)	4.9 (3.3)	0.22	9.6 (2.1)	10.6 (2.1)	0.47
Completed cycles	3.3 (1.9)	4.2 (2.0)	0.45	–	–	–	1.0 (0)	1.0 (−)	–	2.8 (1.8)	3.1 (1.8)	0.14	1.5 (1.4)	2.6 (3.1)	0.49
Intermediate risk	*N* = 3243			*N* = 3765			*N* = 4269			*N* = 1916			*N* = 1251		
Proportion, row %	81.3	18.7		91.2	8.8		66.6	33.4		24.6	75.4		78.7	21.3	
Age, years (SD)	64.1 (13.1)	61.6 (12.1)	− 0.20	63.2 (12.0)	63.5 (12.2)	0.03	70.2 (8.5)	70.0 (8.1)	− 0.03	61.9 (13.3)	68.6 (11.8)	0.53	65.0 (12.7)	66.5 (11.1)	0.13
Sex, female	100.0	100.0	0	46	46.5	0.01	47.3	49.1	0.04	40.0	47.9	0.16	100.0	100.0	0
Comorbidities															
CVD	9.9	7.4	− 0.09	16.5	19	0.07	29.6	31.2	0.04	13.1	15.0	0.05	12.9	17.7	0.13
CKD	6.5	3.8	− 0.12	8.1	6	− 0.08	10.6	11.6	0.03	9.3	9.2	0	6.4	9.8	0.12
COPD	4.1	3.0	− 0.06	5.6	5.4	− 0.01	22.0	22.7	0.02	3.6	5.1	0.07	3.5	4.9	0.07
Diabetes type 2	20.0	19.9	0	23.8	26.6	0.06	24.7	26.6	0.04	18.9	22.6	0.09	15.3	16.2	0.02
Liver disease	2.4	2.5	0.01	8.8	10	0.04	3.8	3.9	0.01	3.0	3.9	0.05	4.6	5.6	0.05
Metastasis	25.6	20.4	− 0.12	53.4	51.7	− 0.04	24.8	25.9	0.03	3.8	3.9	0.01	40.8	39.1	− 0.04
NRH	0.3	0.8	0.08	0.1	0	− 0.05	0.5	0.7	0.03	2.8	2.5	− 0.02	0.4	0.4	− 0.01
Charlson comorbidity index	6.7 (3.8)	6.5 (3.6)	− 0.06	8.4 (3.3)	8.4 (3.5)	0	8.4 (3.4)	8.9 (3.2)	0.14	4.2 (3.0)	4.4 (3.0)	0.05	7.7 (3.3)	7.9 (3.3)	0.07
Completed cycles	2.2 (2.2)	4.9 (2.6)	1.12	5.0 (2.7)	5.1 (2.7)	0.04	2.0 (1.6)	3.0 (1.9)	0.59	3.6 (2.1)	3.8 (2.0)	0.12	2.4 (1.9)	3.9 (1.9)	0.78
Intermediate risk with ≥1 risk factor^a^	*N* = 1638			*N* = 2838			*N* = 3169			*N* = 880			*N* = 784		
Proportion, row %	83.0	17.0		91.3	8.7		66.4	33.6		20.7	79.3		78.7	21.3	
Age, years (SD)	67.8 (11.7)	64.9 (10.8)	− 0.26	64.6 (11.5)	65.0 (11.8)	0.03	71.2 (8.0)	70.6 (7.9)	− 0.08	67.6 (12.2)	71.6 (9.3)	0.37	66.8 (11.8)	67.5 (11.0)	0.07
Sex, female	100.0	100.0	0	45.7	46.0	0.01	46.9	47.6	0.01	42.3	48.7	0.13	100.0	100.0	0
Comorbidities															
CVD	19.3	16.1	− 0.08	21.8	25.4	0.09	40.0	41.8	0.04	34.1	30.9	− 0.07	20.6	28.1	0.18
CKD	12.6	8.2	− 0.14	10.7	8.1	− 0.09	14.3	15.5	0.04	24.2	19.1	− 0.13	10.2	15.6	0.16
COPD	7.9	6.5	− 0.06	7.4	7.3	− 0.01	29.6	30.5	0.02	9.3	10.5	0.04	5.5	7.8	0.09
Diabetes type 2	38.8	43.4	0.09	31.6	35.5	0.08	33.3	35.6	0.05	48.9	46.8	− 0.04	24.5	25.7	0.03
Liver disease	4.6	5.4	0.04	11.7	13.3	0.05	5.1	5.2	0.00	7.7	8.2	0.02	7.3	9.0	0.06
Metastasis	49.6	44.4	− 0.10	70.8	69.0	− 0.04	33.4	34.7	0.03	9.9	8.2	− 0.06	65.2	62.3	− 0.06
NRH	0.4	1.8	0.14	0.2	0	− 0.06	0.6	0.9	0.04	6.6	4.7	− 0.08	0.5	0.6	0.02
Charlson comorbidity index	8.3 (3.4)	8.2 (3.3)	− 0.03	9.0 (2.8)	9.2 (3.0)	0.06	8.8 (3.4)	9.3 (3.2)	0.14	5.6 (3.3)	5.3 (3.2)	− 0.09	8.8 (2.7)	8.7 (3.0)	− 0.04
Completed cycles	2.1 (2.1)	4.8 (2.5)	1.19	5.1 (2.7)	5.1 (2.6)	0.02	2.0 (1.6)	3.1 (1.9)	0.62	3.4 (2.1)	3.7 (2.0)	0.16	2.3 (1.9)	3.9 (2.0)	0.81

Data presented as column %, except for age, Charlson comorbidity index, and completed cycles which are presented as mean (SD), and proportion of patients as row %

^a^Chemotherapy regimen with intermediate FN risk and ≥ 1 patient-level risk factor. Risk factors include patients’ age > 65 years at index date, baseline diagnosis of metastatic disease, diabetes mellitus, CVD (defined as myocardial infarction, heart failure, peripheral vascular disease, or stroke), COPD, liver or renal dysfunction, and HIV/AIDS

*CKD* chronic kidney disease, *COPD* chronic obstructive pulmonary disease, *CVD* cardiovascular disease, *FN* febrile neutropenia, *G-CSF* granulocyte colony–stimulating factor, *HF* heart failure, *HIV/AIDS* human immunodeficiency virus/acquired immune deficiency syndrome; *NRH* neutropenia-related hospitalization, *SD* standard deviation, *Std diff* standardized difference

**Fig. 1:**
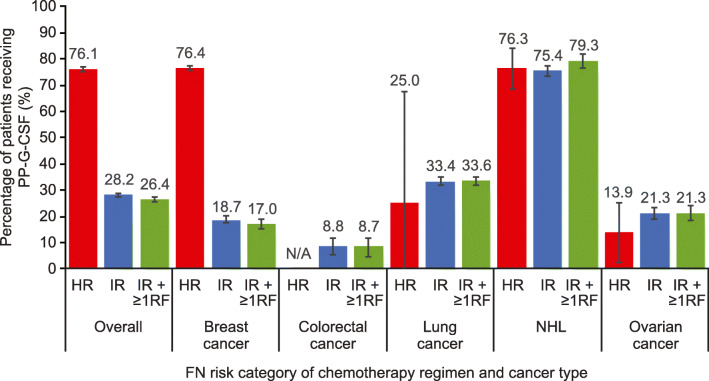
Proportion of eligible patients receiving prophylactic G-CSF in the first cycle, stratified by the FN risk category of the chemotherapy regimen and cancer type. Error bars are 95% confidence intervals. *FN* febrile neutropenia, *HR* chemotherapy regimen with high FN risk, *IR* chemotherapy regimen with intermediate FN risk, *IR + ≥ 1RF* chemotherapy regimen with intermediate FN risk and ≥ 1 patient-level risk factor, *N/A* not applicable because patients diagnosed with colorectal cancer did not receive any chemotherapy regimen with high FN risk, *NHL* non-Hodgkin lymphoma, *PP-G-CSF* primary prophylactic-granulocyte colony–stimulating factor

Proportions of patients receiving prophylactic G-CSF in the first cycle were 76.1%, 28.2%, and 26.4% for patients receiving chemotherapy with high risk, intermediate risk, or intermediate risk for FN with ≥ 1 risk factor, respectively (Fig. 1, Online Resource 8). Prophylactic G-CSF use among patients receiving chemotherapy regimens with intermediate FN risk versus those receiving chemotherapy regimens with intermediate FN risk with ≥ 1 risk factor was similar across the 5 cancer types.

**Fig. 2:**
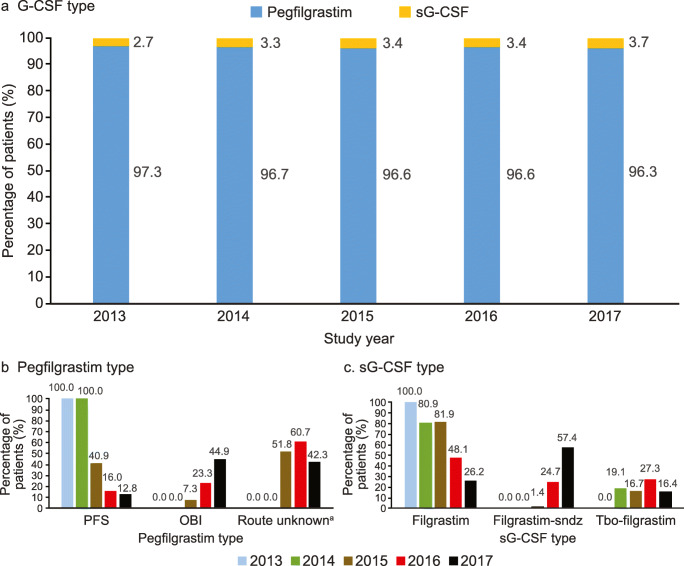
Proportion of prophylactic G-CSF use by type among patients receiving chemotherapy with high/intermediate FN risk and prophylactic G-CSF in the first cycle by calendar year, and by G-CSF type (**a**), Pegfilgrastim type (**b**), and sG-CSF type (**c**). ^a^Route unknown: pegfilgrastim users who could not be classified as pegfilgrastim PFS or OBI. *FN* febrile neutropenia, *G-CSF* granulocyte colony–stimulating factor, *OBI* on-body injector, *PFS* prefilled syringe, *prophylactic G-CSF* primary prophylaxis G-CSF, *sG-CSF* short-acting G-CSF

Among patients receiving prophylactic G-CSF, ≥ 96% were administered pegfilgrastim and this remained unchanged over the study period (Fig. 2, Online Resource 9*).* Prophylactic pegfilgrastim use was similar across patients receiving chemotherapy regimens with high (98.0%), intermediate (94.7%), and intermediate FN risk with ≥ 1 risk factor (94.4%). Use of the OBI formulation increased to 44.9% by 2017 (42.3% of use could not be classified as either PFS or OBI) (Fig. 2). Among patients receiving prophylactic sG-CSFs, biosimilar filgrastim use increased to ≥ 70% by the end of the time period. Filgrastim use decreased from 100% in 2013 to 26.2% in 2017, with an increase in filgrastim-sndz and tbo-filgrastim use (Fig. 2). Overall, there was no major difference between mean number of administrations per cycle for filgrastim (3.2 [SD 2.3]), filgrastim-sndz (3.0 [SD 1.6), and tbo-filgrastim (4.3 [SD 2.5]) (Online Resource 9).

**Fig. 3:**
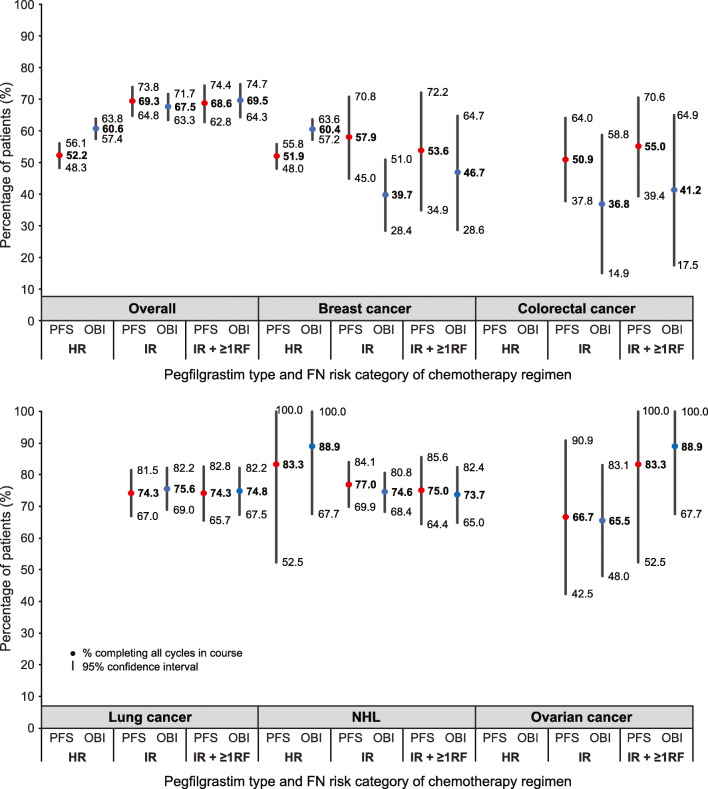
Proportion of patients completing all cycles in their chemotherapy course with prophylactic pegfilgrastim (i.e., persistence), stratified by pegfilgrastim type and FN risk category of the chemotherapy regimen (March 1, 2015, to December 31, 2017). *FN* febrile neutropenia, *HR* chemotherapy regimen with high FN risk, *IR* chemotherapy regimen with intermediate FN risk, *IR + ≥ 1RF* chemotherapy regimen with intermediate FN risk and ≥ 1 patient-level risk factor, *NHL* non-Hodgkin lymphoma, *OBI* pegfilgrastim on-body injector, *PFS* pegfilgrastim prefilled syringe, *prophylactic pegfilgrastim* primary prophylaxis pegfilgrastim

Among cancer patients receiving chemotherapy regimens with high FN risk and prophylactic pegfilgrastim (Fig. 3), 60.6% (95% CI 57.4%, 63.8%) of patients receiving prophylactic pegfilgrastim with the OBI in the first cycle completed all their cycles in their course with the OBI (i.e., persistence); however, only 52.2% (95% CI 48.3%, 56.1%) of patients receiving prophylactic pegfilgrastim with the PFS in the first cycle completed all their cycles in their course with the PFS. These findings were largely driven by the breast cancer population. There were no material differences in completion rates across the two formulations among patients receiving chemotherapy regimens with intermediate FN risk (Online Resource 10).

**Fig. 4:**
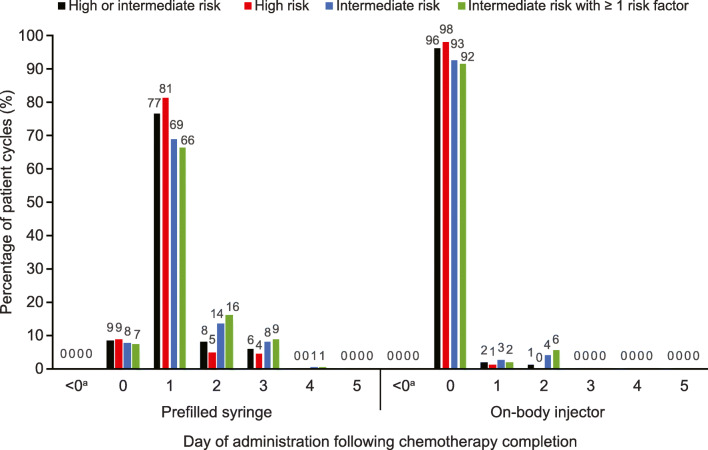
Day of pegfilgrastim administration following chemotherapy completion by mode of delivery and FN risk category of the chemotherapy regimen. On-body injector automatically administers pegfilgrastim at 27 h after application. ^a^Claims appearing before chemotherapy completion date (i.e., day 0) but after chemotherapy initiation were considered primary prophylactic and were 0.2%. *FN* febrile neutropenia

Overall, among all patient cycles receiving pegfilgrastim prophylaxis, the proportion of patient cycles receiving pegfilgrastim on the same day as completion of chemotherapy with high/intermediate FN risk was 9% for pegfilgrastim PFS and 0% for pegfilgrastim OBI (Fig. 4). The proportion of patients receiving pegfilgrastim PFS on the same day was highest for the first cycle (13.2%) and declined in the subsequent cycles to 2.8% in cycle 8 (Online Resource 11)*.*

## Discussion

Using data from a nationally representative population with commercial and Medicare Advantage insurance, we describe the use of G-CSF prophylaxis in 5 cancer types treated with chemotherapy regimens with high/intermediate FN risk in clinical practice. We found that 76% of patients receiving chemotherapy regimens with high FN risk were receiving prophylactic G-CSF, but only 28% of patients receiving regimens with intermediate FN risk received prophylactic G-CSF. Presence of ≥ 1 patient risk factor for FN among patients receiving regimens with intermediate FN risk did not seem to affect the decision to provide prophylaxis. During the study period, > 95% of patients received long-acting G-CSF, with a steady increase in the use of the OBI delivery device. Patients with breast cancer were most likely to receive chemotherapy regimens with high FN risk. In this population, users of the OBI delivery device were more likely to complete all their chemotherapy cycles with pegfilgrastim compared to users of the PFS. During the study period, biosimilar sG-CSF use increased steadily, with filgrastim-sndz accounting for most of the sG-CSF use by the end 2017.

Our results are consistent with prior studies that reported suboptimal use of prophylactic G-CSF [24, 25]. Ramsey and colleagues linked the Western Washington State Surveillance, Epidemiology, and End Results (SEER) registry data with data from 4 major insurers (Medicare, Medicaid, Premera Blue Cross, and Regence Blue Shield) for patients treated for breast cancer, non-small cell lung cancer, and colorectal cancer from 2002 to 2005 [24]. They observed prophylactic G-CSF use of 33% and 10% among patients receiving high risk FN chemotherapy and intermediate FN risk chemotherapy, respectively. In a retrospective cohort study using a 20% Medicare sample from 2007 to 2011, Sosa and colleagues observed that 74% of breast cancer patients and 62% of NHL patients receiving high FN risk chemotherapy received prophylactic G-CSF in the first cycle [25]. In their study, prophylactic G-CSF use among patients receiving intermediate FN risk chemotherapy was 10% for breast cancer and 20% for lung cancer patients. The relatively higher use of prophylactic G-CSF among patients receiving high FN risk chemotherapy observed in our study and in the study by Sosa and colleagues compared to the older study by Ramsey et al. is a reflection of the gradual increase in use of prophylactic G-CSF since 2002, primarily due to the introduction of pegfilgrastim [26].

Uniquely, in our study, we observed that eligible patients not receiving prophylaxis were not dissimilar from patients who received prophylactic G-CSF in terms of age and major comorbidities. The only difference we observed was a higher proportion of metastatic diagnosis among breast cancer patients treated with chemotherapy regimens with intermediate FN risk and not receiving prophylactic G-CSF compared to those receiving G-CSF. Treatment for metastatic patients is usually palliative and focused on improving quality of life, and the evidence of impact of dose intensity on patient outcomes is not conclusive [27]. This may explain the lower proportion of metastatic diagnosis among patients receiving prophylactic G-CSF, which aligns with prior observational studies [28] and shows oncologists’ adherence to American Society of Clinical Oncology guidelines on prophylactic G-CSF use in the metastatic setting [29]. We also observed that patients receiving chemotherapy regimens with intermediate FN risk chemotherapy and ≥ 1 risk factor did not have a higher use of G-CSF compared to patients receiving chemotherapy regimens with intermediate FN risk. This finding demonstrates a need for wider dissemination of the NCCN® guidelines that recommend consideration of G-CSF use among patients receiving chemotherapy regimens with intermediate risk for FN with ≥ 1 risk factor.

Patients who continue to be persistent with pegfilgrastim in subsequent cycles of a chemotherapy course have a lower risk for developing FN than those who discontinue early [30, 31]. In a non-inferiority randomized trial of early breast cancer patients receiving a chemotherapy regimen with high FN risk, Aarts and colleagues [30] found a more than 3-fold higher incidence of FN (36% versus 10%) among patients assigned to receive pegfilgrastim for the first 2 cycles only compared to those who received pegfilgrastim through all 6 cycles. Consequently, it is important to understand if novel pegfilgrastim delivery devices such as the OBI [17] offer any persistent benefit to high FN risk patients compared to the traditional PFS delivery. In breast cancer patients receiving chemotherapy regimens with high FN risk, we observed an 8.5% increase in patients who completed all their cycles with pegfilgrastim support if they started with pegfilgrastim OBI (60.4%) compared to pegfilgrastim PFS (51.9%).

Similar to our findings, prior studies reported considerable proportions of patients receiving same-day administration of PFS [32, 33]. In a retrospective cohort study using 2 large commercial claims databases from 2003 to 2011, Weycker and colleagues reported that among primary solid tumor and NHL patients receiving pegfilgrastim PFS prophylaxis (*n* = 37,095), PFS was administered on the same day as chemotherapy completion in 12% of the cycles [32]. A follow-up study by the same investigators using the same databases from 2010 to 2015 but restricted to chemotherapy regimens with high/intermediate risk for FN reported that 8% of the 21,7273 cycles received pegfilgrastim on the same day as chemotherapy completion [33]. In our study, we observed that 9% of all cycles received pegfilgrastim PFS on the same day, with almost no pegfilgrastim use via OBI administration occurring on the same day. Note that the claim for OBI application appears in the database on the same day as chemotherapy completion and the automatic administration of pegfilgrastim occurs 27 h after the application. Uniquely, we observed same-day administration was highest in the first cycle and decreased in subsequent cycles in our study. Given the increased risk for FN associated with same-day administration of pegfilgrastim [32–34] and the increased risk for FN in the first cycle compared to subsequent cycles [35], an automated OBI that can deliver pegfilgrastim in the ideal time window can alleviate travel burden and fulfill an unmet need for these patients. Future studies are needed to quantify the FN risk reduction resulting from the improved persistence and appropriately timed pegfilgrastim administration offered by novel drug delivery devices.

In recent years, biosimilars for both filgrastim (filgrastim-sndz and filgrastim-aafi) [12, 13] and pegfilgrastim (pegfilgrastim-jmdb and pegfilgrastim-cbqv) [15, 16] and an original sG-CSF (tbo-filgrastim) [14] have been approved and marketed in the USA. The increasing adoption of filgrastim biosimilars including filgrastim-sndz and tbo-filgrastim observed in our study aligns with the timeline and proportions presented in previous publications using Anthem administrative claims [36] and Medicare databases [37]. The (mean [SD]) administration days of prophylactic filgrastim (3.2 [2.3]) observed in our study was higher than that reported by Schwartzberg et al. [38] (2.1 [1.4]), but lower than that reported by Naeim et al. [39] (4.8 [3.3]). Schwartzberg et al. used information from the Optum™ database between March 2015 and June 2016 and did not restrict their study to NCCN®-recommended chemotherapy regimens with high/intermediate FN risk; the lower mean observed in their study could be a result of capturing all possible instances of prophylactic filgrastim use. Nevertheless, patients in real-world settings are receiving shorter duration of sG-CSF compared to the 10–11 days suggested by non-inferiority studies comparing filgrastim with pegfilgrastim [40, 41]. To our knowledge, this is the first study to describe the increasing use of novel delivery devices such as OBI among patients receiving chemotherapy regimens with high/intermediate FN risk. By 2017, of all patients that received prophylactic pegfilgrastim, 12.8% received PFS, 44.9% received OBI, and 42.3% received pegfilgrastim by an unknown mode of delivery.

Several limitations need to be considered for our study. First, the administrative claims database we used did not include patient-level risk factors for FN, such as persistent neutropenia, bone marrow involvement by tumor, recent surgery and/or open wounds, or poor performance status. Other determinants of prophylactic G-CSF use, such as practice-level guidelines, therapeutic intent, or patient preferences, were also unavailable in the claims database. We included type 2 diabetes, cardiovascular disease, chronic obstructive pulmonary disease, and baseline metastasis based on expert guidance as patient-level risk factors, although these are not included in the NCCN® list of risk factors. Nevertheless, the presence of ≥ 1 risk factor did not increase an eligible patient’s likelihood of receiving prophylactic G-CSF compared to a broader subgroup of patients receiving a chemotherapy regimen with intermediate FN risk. Secondly, we were unable to accurately discriminate a portion of pegfilgrastim users as PFS or OBI after the launch date of the OBI (March 1, 2015) because of the lack of a specific J-code for the OBI. If the ratio of OBI:PFS in 2017 in the pegfilgrastim route unknown subgroup is similar to the ratio of the pegfilgrastim route known subgroup (45:13), OBI would make up 78% of all pegfilgrastim administrations in 2017. Thirdly, chemotherapy dose is not described in the claims database, which may have resulted in inclusion of patients with reduced dose intensity (i.e., ineligible for prophylactic G-CSF) in the subgroups, thus leading to an underestimation of prophylactic G-CSF use. Assuming chemotherapy dose reduction occurs more frequently in later cycles, we expect minimal underestimation of prophylactic G-CSF use from our definition in the first cycle in this study. Fourthly, we included patients only until August 31, 2017. This limited the follow-up period to capture treatment in later cycles for patients entering the cohort near the end of the inclusion period, effectively reducing the precision of our estimates. While this study provides important data on chemotherapy and prophylactic G-CSF use in patients with various tumors, it was not able to determine the reasons for cycle interruptions. It is hypothesized that a non-trivial proportion may be due to FN but further research is needed to better understand this multi-factorial process. Finally, the population in this study was obtained from a nationally representative insurer but the results may not generalize to patients with Medicare or for patients without insurance.

In conclusion, the results from this nationally representative database demonstrate that cancer patients at high risk for FN may be vulnerable because of under- or mistimed use of prophylactic G-CSF. Future studies are required to understand the reasons for G-CSF underutilization and quantify the FN risk reduction resulting from improved persistence and appropriately timed dosing of pegfilgrastim administered through a novel delivery device compared to pegfilgrastim administered through the PFS.

## Electronic supplementary material

ESM 1 (PDF 510 kb)
