# Supplementary material for: Patterns of granulocyte colony–stimulating factor prophylaxis in patients with cancer receiving myelosuppressive chemotherapy
Source: Support Care Cancer. 2020 Jan 10;28(9):4413–24. doi: 10.1007/s00520-020-05295-2 (PMC7378111; doi:10.1007/s00520-020-05295-2)
Supplement: Supplementary file 1 — (PDF 510 kb) [file 520_2020_5295_MOESM1_ESM.pdf]

Supplemental material

Title:

Patterns of granulocyte colony-stimulating factor prophylaxis in patients with cancer receiving myelosuppressive chemotherapy

Authors:

Prasad L. Gawade,[1] Shuling Li,[2] David Henry,[3] Nancy Smith,[4] Rajesh Belani,[5] Michael A. Kelsh,[1] Brian D. Bradbury[1]

Affiliations:

[1]Center for Observational Research, Amgen Inc., Thousand Oaks, CA, USA

[2]Chronic Diseases Research Group, Hennepin Healthcare Research Institute, Minneapolis, MN, USA

[3]Department of Medicine, University of Pennsylvania, Philadelphia, PA, USA

[4]DOCS Global, Watham, MA, USA

[5]US Medical Affairs, Amgen Inc., Thousand Oaks, CA, USA

Corresponding author: Prasad L. Gawade, PhD, Amgen Inc., Center for Observational Research, One Amgen Center Drive, Thousand Oaks, CA 91320, USA

Phone: 1 (805) 477 0741

Email: pgawade@amgen.com

Journal: *Supportive Care in Cancer*

Supplemental material: ESM_1; 12 online resources (Supplemental Methods, 11 supplemental tables)

Online Resource 1 List of chemotherapy regimens by cancer type and risk of developing FN, as defined by the NCCN® 2017 v2. Guidelines [1]

Cancer type	Risk of developing FN<br>High (>20%)	 Intermediate (10-20%)
Breast cancer	TC (docetaxel + cyclophosphamide)<br>Dose-dense AC → T (doxorubicin, cyclophosphamide, paclitaxel)<br>TAC (docetaxel, doxorubicin, cyclophosphamide)<br>TCH (docetaxel, carboplatin, trastuzumab)	Docetaxel every 21 days<br>CMF classic (cyclophosphamide, methotrexate, fluorouracil)<br>AC (doxorubicin, cyclophosphamide) + sequential docetaxel<br>FEC (fluorouracil, epirubicin, cyclophosphamide) + sequential docetaxel<br>Paclitaxel every 21 days
Colorectal cancer	-	FOLFOX (fluorouracil, leucovorin, oxaliplatin)
Lung cancer	Topotecan	Etoposide/carboplatin<br>Cisplatin/paclitaxel<br>Cisplatin/vinorelbine<br>Cisplatin/docetaxel<br>Cisplatin/etoposide<br>Carboplatin/paclitaxel<br>Carboplatin/docetaxel<br>Docetaxel<br>Paclitaxel
Non-Hodgkin lymphoma	ICE (ifosfamide, carboplatin, etoposide)<br>RICE (rituximab, ifosfamide, carboplatin, etoposide)<br>CHOP-14 (cyclophosphamide, doxorubicin, vincristine, prednisone)<br>MINE (mesna, ifosfamide, novantrone, etoposide)<br>DHAP (dexamethasone, cisplatin, cytarabine)<br>ESHAP (etoposide, methylprednisolone, cisplatin, cytarabine)<br>HyperCVAD + rituximab (cyclophosphamide, vincristine, doxorubicin, dexamethasone + rituximab)	GDP (gemcitabine, dexamethasone, cisplatin)<br>GDP (gemcitabine, dexamethasone, cisplatin) + rituximab<br>CHOP + rituximab (cyclophosphamide, doxorubicin, vincristine, prednisone + rituximab)

	EPOCH (etoposide, prednisone, vincristine, cyclophosphamide, doxorubicin)<br>EPOCH (etoposide, prednisone, vincristine, cyclophosphamide, doxorubicin) + IT chemotherapy	
Ovarian cancer	Topotecan<br>Docetaxel	Carboplatin/docetaxel<br>Carboplatin/paclitaxel

FN febrile neutropenia, *IT* intrathecal

Online Resource 2 Study design for annual patient cohorts who initiated myelosuppressive chemotherapy. Eligible patients had breast, colorectal, lung, or ovarian cancer or NHL and had initiated myelosuppressive chemotherapy in each year from January 1, 2012 to August 31, 2017. Demographics, clinical characteristics, and continuous enrollment were assessed in the baseline period. G-CSF use and persistence and timing of PFS administration were assessed in the follow-up period. The study follow-up period started on the index date and ended on the earliest date of the last day of the last cycle of chemotherapy course, last day of cycle 8 of the chemotherapy course, occurrence of an FN-related hospitalization (≥1 inpatient diagnosis claim in any position), bone marrow or stem cell transplant or radiation therapy (≥1 inpatient or outpatient diagnosis/procedure claim in any position), death, disenrollment from commercial plan, or December 31, 2017

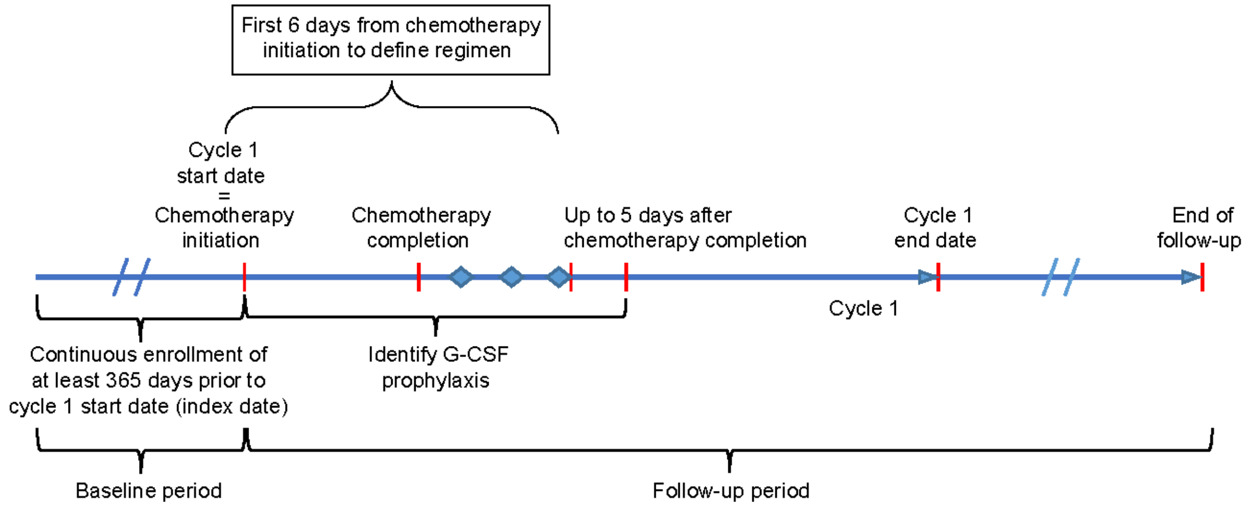

FN febrile neutropenia, *G-CSF* granulocyte colony-stimulating factor, *NHL* non-Hodgkin lymphoma, *PFS* prefilled syringe

Online Resource 3 List of codes used to identify censoring events, baseline comorbidities, and patient-level risk factors

Comorbidity	ICD-9-CM	ICD-10-CM
Chronic kidney disease	V42.0, V45.1x, V45.11, V45.12, V56, V56.0, V56.1, V56.2,V56.3, V56.31, V56.32, V56.8, 403.0, 403.00, 403.01, 403.11, 403.91, 404.01, 404.02, 404.03, 404.11, 404.12, 404.13, 404.91, 404.92, 404.93, 582, 582.0, 582.1, 582.2, 582.4, 582.8, 582.81, 582.89, 582.9, 583.89, 583.9, 585.1, 585.2, 585.3, 585.4, 585.5, 585.6, 585.9, 587, 588, 588.0, 588.1, 588.8, 588.81, 588.89, 588.9	I13.0, I13.I, I13.10, I13.11, I13.2, I12, I12.0, I12.9, N03.1, N03.2, N03.3, N03.4, N03.5, N03.6, N03.7, N03.8, N03.9, N13.9, N18, N18.1, N18.2, N18.3, N18.4, N18.5, N18.6, N18.9, N19, N25, N25.0, N25.1, N25.8, N25.81, N25.89, N25.9, Z49, Z49.0, Z49.01, Z49.02, Z49.3, Z49.31, Z49.32, Z94.0, Z99.2
Chronic obstructive pulmonary disease	416.8, 416.9, 506.4, 508.1, 508.8	I27.8, I27.81, I27.82, I27.89, I27.9, J40, J41, J41.0, J41.1, J41.8, J42, J43, J43.0, J43.1, J43.2, J43.8, J43.9, J44, J44.0, J44.1, J44.9, J47.0, J47.1, J47.9, J60, J61, J62, J62.0, J62.8, J63, J63.0, J63.1, J63.2, J63.3, J63.4, J63.5, J63.6, J64, J66, J66.0, J66.1, J66.2, J66.8, J67, J67.0, J67.1, J67.2, J67.3, J67.4, J67.5, J67.6, J67.7, J67.8, J67.9, J68.4, J70.1, J70.3
Type 2 diabetes	250.00, 250.02, 250.10, 250.12, 250.20, 250.22, 250.30, 250.32, 250.4, 250.40, 250.41, 250.42, 250.43, 250.50, 250.52, 250.60, 250.62, 250.70, 250.72, 250.80, 250.82, 250.90, 250.92	E11, E11.0, E11.00, E11.01, E11.3, E11.31, E11.311, E11.319, E11.32, E11.321, E11.329, E11.33, E11.331, E11.339, E11.34, E11.341, E11.349, E11.35, E11.351, E11.359, E11.36, E11.39, E11.4, E11.40, E11.41, E11.42, E11.43, E11.44, E11.49, E11.5, E11.51, E11.52, E11.59, E11.6, E11.61, E11.610, E11.618, E11.62, E11.620, E11.621, E11.622, E11.628, E11.63, E11.630, E11.638, E11.64, E11.641, E11.649, E11.65, E11.69, E11.8, E11.9, E10.22, E11.22, E11.2, E11.21, E11.29
Heart failure	402.01, 402.11, 402.91, 398.91, 404.01, 404.03, 404.11, 404.13, 404.91, 404.93, 428, 428.0, 428.1, 428.2, 428.20, 428.21, 428.22, 428.23, 428.3, 428.30, 428.31, 428.32, 428.33, 428.4, 428.40, 428.41, 428.42, 428.43, 428.9	I11.0, I09.81, I50, I50.2, I50.20, I50.21, I50.22, I50.23, I50.3, I50.30, I50.31, I50.32, I50.33, I50.4, I50.40, I50.41, I50.42, I50.43, I50.8, I50.81, I50.810, I50.811, I50.812, I50.813, I50.814, I50.82, I50.83, I50.84, I50.89, I50.9, I97.13, I97.130, I97.131, T86.22, T86.32, I13.0, I13.2

Comorbidity	ICD-9-CM	ICD-10-CM
HIV/AIDS	042, 042.0, 042.1, 042.2, 042.9, 043, 043.1, 043.2, 043.3, 043.9, 044, 044.0, 044.9	B20, B20.0, B20.1, B20.2, B20.3, B20.4, B20.5, B20.6, B20.7, B20.8, B20.9, B21, B21.0, B21.1, B21.2, B21.3, B21.7, B21.8, B21.9, B22, B22.0, B22.1, B22.2, B22.7, B23.0, B23.1, B23.2, B23.8, B24, B97.35, O98.7, O98.71, O98.711, O98.712, O98.713, O98.719, O98.72, O98.73, Z21
Infection	002, 002.0-002.9, 003.0-003.2, 003.20-003.29, 003.3-003.9, 004, 004.0-004.9, 008, 008.0, 008.00-008.09, 008.1-008.4, 008.41-008.49, 008.5, 008.6, 008.61-008.69, 008.8, 034, 034.0, 034.1, 035, 036, 036.0-036.4, 036.40-036.43, 036.8, 036.81-036.89, 039, 039.0-039.9, 040, 040.0-040.4, 040.41, 040.42, 040.8, 040.81-040.89, 041, 041.12, 041.19, 041.2-041.4, 041.41-041.49, 041.5-041.8, 041.81-041.89, 041.9, 101, 112.8, 112.81-112.85, 114, 114.1-114.9, 115, 115.01-115.09, 115.1, 115.11-115.19, 115.9, 115.91-115.99, 116, 116.0-116.2, 117.0-117.9, 118, 320, 320.0-320.8, 320.81-320.89, 320.9, 321, 321.0-321.8, 324, 324.0-324.9, 360.0, 360.00-360.04, 380.1, 380.10-380.16, 380.2, 380.22, 380.23, 383.0, 383.00-383.02, 383.1, 383.2, 383.20-383.22, 376, 376.0, 376.00, 420.99, 421, 421.0, 421.1, 461, 461.0-461.9, 462, 463, 475, 481, 482.0-482.3, 482.30-482.39, 482.4, 482.40-482.49, 482.8, 482.81-482.89, 485, 486, 491.21, 494, 494.0, 494.1, 510, 510.0, 510.9, 513, 513.0, 513.1, 522.5, 522.7, 526.4, 527.3, 528.3, 540, 540.0-540.9, 541, 542, 562.01, 562.03, 562.11, 562.13, 566, 567.0-567.2,	A01, A01.00, A01.1-A01.4, A02.0-A02.2, A02.20-A02.29, A02.8, A02.9, A03.0-A03.9, A04.0-A04.9, A08.0, A08.11, A08.19, A08.2, A08.31-A08.39, A08.8, A22.7, A26.7, A32.7, A38.9, A39.0, A39.1, A39.4, A39.50-A39.53, A39.81-A39.89, A40, A40.0-A40.9, A41, A41.52, A41.9, A42.0-A42.7, A42.81-A42.89, A42.9, A43.8, A43.9, A46, A48.0-A48.3, A48.51, A48.52, A48.8, A54.86, A69.0, A69.1, B37.5-B37.7, B37.81-B37.84, B38.0-B38.9, B39.3-B39.9, B40.9, B41.0, B41.9, B42.0-B42.7, B42.9, B43.9, B44.9, B45.0, B45.1, B45.7, B45.9, B46.9, B47.0-B47.9, B48.0-B48.2, B48.8, B49, E83.2, G02, G06.0-G06.2, H05.00, H32, H44.0, H44.009, H44.01, H44.011-H44.019, H44.02, H44.021-H44.029, H44.09, H60.00, H60.10, H60.20, H60.319, H60.329, H60.339, H60.399, H60.509, H60.519, H60.529, H60.549, H60.559, H60.599, H60.60, H60.8X1, H60.90, H61.93, H62.40, H62.8X1, H70.009, H70.01, H70.011-H70.019, H70.091-H70.099, H70.10, H70.209, H70.219, H70.229, I30.8, I32, I33.0, I39, I96, J01.00, J01.10, J01.20, J01.30, J01.40, J01.90, J02.0, J02.9, J03.00, J03.90, J13, J14, J15.0, J15.1, J15.20, J15.211, J15.212, J15.29, J15.3-J15.8, J18.0, J18.1, J18.9, J36, J44.1, J47.1, J47.9, J85.0-J85.3, J86.0, J86.9, K04.6, K04.7, K12.2, K35.2, K35.3, K35.80, K35.89, K36, K37, K57.12, K57.13, K57.32, K57.33, K61.0, K61.1, K61.3, K65.0-K65.9, K67, K68.12, K68.19, K68.9, K81.0-K81.9, K85.7, K90.81, K94.02, K94.12, L02.02, L02.03, L02.12, L02.13, L02.221-L02.229, L02.231-L02.239, L02.33, L02.429, L02.439, L02.529, L02.539, L02.629, L02.639, L02.821, L02.828, L02.831, L02.838, L02.92, L02.93, L03.019, L03.029, L03.039, L03.049, L03.119, L03.129, L03.211, L03.211, L03.212, L03.221, L03.222, L03.317, L03.319, L03.329, L03.811, L03.818, L03.891, L03.898, L03.90, L03.91, L04.9, L05.01, L05.02, L08.0, L08.1, L08.89, L08.9, L88, L98.0, M00.039, M00.049, M00.059, M00.069, M00.079, M00.09, M00.129, M00.139, M00.149, M00.159, M00.169,

Comorbidity	ICD-9-CM	ICD-10-CM
	567.21-567.29, 567.3, 567.31-567.39, 567.8, 567.81-567.89, 567.9, 575.0, 575.1, 575.10-575.12, 590.0, 590.00, 590.01, 590.1, 590.10, 590.11, 590.2, 590.3, 590.8, 590.80, 590.81, 590.9, 599.0, 601.0-601.2, 675.1, 675.10-675.14, 680, 680.0-680.9, 681, 681.0, 681.00, 681.1, 681.10, 682, 682.0-682.9, 683, 685, 685.0, 686, 686.0, 686.00-686.09, 686.1-686.9, 711, 711.0, 711.00-711.09, 728.86, 730, 730.0, 730.00-730.09, 730.1, 730.10-730.19, 730.2, 730.20-730.29, 730.3, 730.30-730.39, 730.7, 730.70-730.79, 730.8, 730.80, 730.81, 785.4, 785.52, 790.7, 958.3, 995.91, 995.92, 996.6, 996.60-996.69, 998.5, 998.51, 998.59, 999.3, 999.31-999.39	M00.179, M00.19, M00.229, M00.239, M00.249, M00.259, M00.269, M00.279, M00.29, M00.829, M00.839, M00.849, M00.859, M00.869, M00.87, M00.871, M00.872, M00.879, M00.88, M00.89, M00.9, M46.20, M60.009, M86.10, M86.119, M86.129, M86.139, M86.149, M86.159, M86.169, M86.179, M86.18, M86.19, M86.20, M86.219, M86.229, M86.239, M86.249, M86.259, M86.269, M86.28, M86.29, M86.60, M86.619, M86.629, M86.639, M86.642, M86.659, M86.669, M86.679, M86.68, M86.69, M86.9, M89.60, M89.619, M89.629, M89.639, M89.649, M89.659, M89.679, M89.68, M89.69, M90.80, M90.819, N10, N11.0, N11.8¸N12, N15.1, N15.9, N16, N28.84-N28.86, O91.111-O91.119, O91.12, R65.2, R65.20, R65.21, R78.81, T80.211A, T80.212A, T80.219A, T80.22XA, T80.29XA, T82.6XXA, T82.7XXA, T79.XXA, T83.51XA, T83.59XA, T83.6XXA, T84.50XA, T84.60XA, T84.7XXA, T84.7XXD, T84.7XXS, T85.7, T85.71XA, T85.79XA, T85.79XD, T85.79XS, T85.9, T88.0XXA
Liver disease	456.0, 456.2, 572.0, 572.1, 572.2, 572.3, 572.4, 572.5, 572.6, 572.7, 572.8, 570, 571.0, 571.1, 571.2, 571.3, 571.4, 571.40, 571.41, 571.42, 571.49, 571.5, V42.7, 571.6, 571.8, 571.9	B18.0, B18.1, B18.2, B18.8, B18.9, K73.0, K73.1, K73.2, K73.8, K73.9, K74.0, K74.1, K74.2, K74.3, K74.4, K74.5, K74.6, K74.60, K74.69, K70, K70.0, K70.1, K70.10, K70.11, K70.2, K70.3, K70.9, K70.4, K70.40, K76.6, K76.7, K70.41, K71.3, K71.4, K71.5, K71.7, K72.1, K72.9, K73, K74, K75, K75.0, K75.1, K75.2, K75.3, K75.4, K75.8, K75.81, K75.89, K75.9, K76, K76.0, K76.1, K76.2, K76.3, K76.4, K76.5, K76.8, K76.81, K76.89, K76.9, K70.30, K70.31, I85.0, I85.9, I86.4, I98.2, K70.9, K73.0, K73.1, K73.2, K73.8, K73.9, K74.0, K74.1, K74.2, K70.3, K74.3, K74.4, K74.5, K74.6, K74.60, K74.69
Metastasis	196, 196.0, 196.1, 196.2, 196.3, 196.5, 196.6, 196.8, 196.9, 197, 197.0, 197.1, 197.2, 197.3, 197.4, 197.5, 197.6, 197.7, 197.8, 198, 198.0, 198.1, 198.2, 198.3, 198.4, 198.5, 198.6, 198.7, 198.8, 198.81, 198.82, 198.89, 199, 199.0, 199.1, 199.2	C77.0, C77.1, C77.2, C77.3, C77.4, C77.5, C77.8, C77.9, C78, C78.0, C78.00, C78.01, C78.02, C78.1, C78.2, C78.3, C78.30, C78.39, C78.4, C78.5, C78.6, C78.7, C78.8, C78.80, C78.89, C78.90, C79, C79.0, C79.00, C79.01, C79.02, C79.1, C79.10, C79.11, C79.19, C79.2, C79.3, C79.31, C79.32, C79.4, C79.40, C79.49, C79.5, C79.51, C79.52, C79.6, C79.60, C79.61, C79.62, C79.7, C79.70, C79.71, C79.72, C79.8, C79.81, C79.82, C79.89, C79.9, C80.0, C80.1, C80.2

Comorbidity	ICD-9-CM	ICD-10-CM
Myocardial infarction	410, 410.02, 410.12, 410.22, 410.32, 410.42, 410.52, 410.62, 410.72, 410.82, 410.92, 410.0, 410.00, 410.01, 410.1, 410.10, 410.11, 410.2, 410.20, 410.21, 410.3, 410.30, 410.31, 410.4, 410.40, 410.41, 410.5, 410.50, 410.51, 410.6, 410.60, 410.61, 410.7, 410.70, 410.71, 410.8, 410.80, 410.81, 410.9, 410.90, 410.91, 412	I21, I21.0, I21.01, I21.02, I21.09, I21.1, I21.11, I21.19, I21.2, I21.21, I21.29, I21.3, I21.4, I25.2
Neutropenia hospitalization	288.0, 288.00, 288.01, 288.02, 288.03, 288.04, 288.09	D70.0, D70.1, D70.2, D70.3, D70.4, D70.8, D70.9
Organ transplantation	V42, V42.1, V42.2, V42.3, V42.4, V42.5, V42.6, V42.8, V42.81, V42.82, V42.83, V42.84, V42.89, V42.9	Z94.0, Z94.1, Z95.3, Z94.5, Z94.6, Z94.7, Z94.2, Z94.4, Z94.81, Z94.84, Z94.83, Z94.82, Z94.89, Z94.9
Peripheral vascular disease	443.8, 443.81, 443.82, 443.89, 443.9, 444, 444.0, 444.01, 444.09, 444.1, 444.2, 444.21, 444.22, 444.8, 444.81, 444.89, 444.9, 451, 451.0, 451.1, 451.11, 451.19, 451.2, 451.8, 451.81, 451.82, 451.83, 451.84, 451.89, 451.9, 452, 453, 453.0-453.4, 453.40-453.42, 453.5, 453.50-453.52, 453.6, 453.7, 453.71-453.79, 453.8, 453.81-453.89, 453.9, 557, 557.0, 557.1, 557.9, 415, 415.0, 415.1, 415.11-415.19	I73, I73.0, I73.00, I73.01, I73.1, I73.8, I73.81, I73.89, I73.9, I74, I74.0, I74.01, I74.09, I74.10, I74.11, I74.19, I74.1-I74.9, I80, I80.0, I80.00, I80.01-I80.03, I80.1, I80.10, I80.11-I80.13, I80.2, I80.201-I80.209, I80.21, I80.211-I80.219, I80.22, I80.221-I80.229, I80.23, I80.231-I80.239, I80.29, I80.291-I80.299, I80.3, I80.8, I80.9, I81, I82, I82.0, I82.1, I82.2, I82.21, I82.210, I82.211, I82.22, I82.220, I82.221, I82.29, I82.290, I82.291, I82.3, I82.4, I82.40, I82.401-I82.409, I82.41, I82.411-I82.419, I82.42, I82.421-I82.429, I82.43, I82.431-I82.439, I82.44, I82.441-I82.449, I82.49, I82.491-I82.499, I82.4Y, I82.4Y1-I82.4Y9, I82.4Z, I82.4Z1-I82.4Z9, I82.5, I82.50, I82.501-I82.509, I82.51, I82.511-I82.519, I82.52, I82.521-I82.529, I82.53, I82.531-I82.539, I82.54, I82.541-I82.549, I82.59, I82.591-I82.599, I82.5Y, I82.5Y1-I82.5Y9, I82.5Z, I82.5Z1-I82.5Z9, I82.6, I82.60, I82.601-I82.609, I82.61, I82.611-I82.619, I82.62, I82.621-I82.629, I82.7, I82.70, I82.701-I82.709, I82.71, I82.711-I82.719, I82.72, I82.721-I82.729, I82.8, I82.81, I82.811-I82.819, I82.89, I82,890, I828.91, I82.9, I82.90, I82.91, I82.A, I82.A1, I82.A11-I82.A19, I82.A2, I82.A21-I82.A29, I82.B, I82.B1, I82.B11-I82.B19, I82.B2, I82.B21-I82.B29, I82.C, I82.C1, I82.C11-I82.C19, I82.C2, I82.C21-I82.C29, K55.0, K55.1, K55.9, I26, I26.0 I26.01-I26.09, I26.9, I26.90-I26.99

Comorbidity	ICD-9-CM	ICD-10-CM
Radiation therapy	V58.0; Procedure codes: 92.20-92.39, HCPCS: G0256, G0261, G0173, G0251, G0339, G0340; CPT: 77401-77499, 77520-77525, 77750-77799, 77371-77373, 77385, 77386, 77387	Z51.0; Procedure codes: D00-D02, D70-D72, D7Y, D80-D82, D8Y, D90-D92, D9Y, DB0-DB2, DBY, DD0-DD2, DDY, DF0-DF2, DFY, DG0-DG2, DGY, DH0-DH2, DHY, DM0-DM2, DMY, DP0-DP2, DPY, DT0-DT2, DTY, DU0-DU2, DUY, DV0-DV2, DVY, DW0-DW2, DWY,
Stroke	430, 431, 433.00-433.91, 434.00, 434.01, 434.10, 434.11, 434.90, 434.91, 435, 435.0-435.3, 435.8, 435.9, 436, 437, 437.0-437.9, 438, 438.0, 438.1, 438.10-438.14, 438.19, 438.2, 438.20-438.22, 438.3, 438.30-438.32, 438.4, 438.40-438.42, 438.5, 438.50-438.53, 438.6, 438.7, 438.8, 438.81-438.85, 438.89, 438.9	I60, I60.0, I60.00-I60.02, I60.1, I60.10-I60.12, I60.2, I60.20-I60.22, I60.3, I60.30-I60.32, I60.4, I60.5, I60.50-I60.52, I60.6-I60.9, I61, I61.0-I61.9, I63, I63.0, I63.00-I63.019, I63.02, I63.03, I63.031-I63.039, I63.09, I63.1, I63.10, I63.11, I63.111-I63.119, I63.12, I63.13, I63.131-I63.139, I63.19, I63.2, I63.20, I63.21, I63.211-I63.219, I63.22, I63.23, I63.231-I63.239, I63.29, I63.3, I63.30, I63.31, I63.311-I63.319, I63.32, I63.321-I63.329, I63.33, I63.331-I63.339, I63.34, I63.341-I63.349, I63.39, I63.4, I63.40, I63.41, I63.411-I63.419, I63.42, I63.421-I63.429, I63.43, I63.431-I63.439, I63.44, I63.441-I63.449, I63.49, I63.5, I63.50, I63.51, I63.511-I63.519, I63.52, I63.521-I63.529, I63.53, I63.531-I63.539, I63.54, I63.541-I63.549, I63.59, I63.6, I63.8, I63.9, I67.0, I67.81-I67.83, I67.89, 167.9
Stem-cell or bone marrow transplant	V42.81, V42.82; Procedure codes: 41.0, 41.00, 41.01, 41.02, 41.03, 41.04, 41.05, 41.06, 41.07, 41.08, 41.09	Z94.81, Z94.84; Procedure codes: 30230G0, 30233G0, 30240G0, 30243G0, 30250G0, 30253G0, 30260G0, 30263G0, 30230G1, 30233G1, 30240G1, 30243G1, 30250G1, 30253G1, 30260G1, 30263G1, 30230Y0, 30233Y0, 30240Y0, 30243Y0, 30250Y0, 30253Y0, 30260Y0, 30263Y0, 30230Y1, 30233Y1, 30240Y1, 30243Y1, 30250Y1, 30253Y1, 30260Y1, 30263Y1, 30230X0, 30233X0, 30240X0, 30243X0, 30250X0, 30253X0, 30260X0, 30263X0, 30230X1, 30233X1, 30240X1, 30243X1, 30250X1, 30253X1, 30260X1, 30263X1

HIV/AIDS human immunodeficiency virus infection and acquired immune deficiency syndrome, *ICD-9-CM/ICD-10-CM* International Classification of Diseases-9th/10th Revision Clinical Modification

Online Resource 4 ICD-9-CM/ICD-10-CM diagnosis codes used to identify cancer types

Diagnosis	ICD-9-CM	ICD-10-CM
Breast cancer	174, 174.0, 174.1, 174.2, 174.3, 174.4, 174.5, 174.6, 174.8, 174.9	C50, C50.0, C50.01, C50.011, C50.012, C50.019, C50.1, C50.11, C50.111, C50.112, C50.119, C50.2, C50.21, C50.211, C50.212, C50.219, C50.3, C50.31, C50.311, C50.312, C50.319, C50.4, C50.41, C50.411, C50.412, C50.419, C50.5, C50.51, C50.511, C50.512, C50.519, C50.6, C50.61, C50.611, C50.612, C50.619, C50.8, C50.81, C50.811, C50.812, C50.819, C50.9, C50.91, C50.911, C50.912, C50.919
Colorectal cancer	153, 153.0, 153.1, 153.2, 153.3, 153.4, 153.5, 153.6, 153.7, 153.8, 153.9, 154, 154.0, 154.1, 154.8	C18, C18.0, C18.1, C18.2, C18.3, C18.4, C18.5, C18.6, C18.7, C18.8, C18.9, C19, C20, C21.2, C21.8
Lung cancer	162.2, 162.3, 162.4, 162.5, 162.8, 162.9, 162.0, 163, 163.0, 163.1, 163.8, 163.9, 162.3, 162.4, 162.5, 162.8, 162.9	C33, C34, C34.0, C34.00, C34.01, C34.02, C34.1, C34.10, C34.11, C34.12, C34.2, C34.3, C34.30, C34.31, C34.32, C34.8, C34.80, C34.81, C34.82, C34.9, C34.90, C34.91, C34.92, C38.4, C7A.090
Non-Hodgkin lymphoma	200, 200.0, 200.00-200.08, 200.1, 200.10-200.18, 200.2, 200.20-200.28, 200.3, 200.30-200.38, 200.4, 200.40-200.48, 200.5, 200.50-200.58, 200.8, 200.80-200.88, 202, 202.00-202.08, 202.1, 202.10-202.18, 202.2, 202.20-202.28, 202.3, 202.30-202.38, 202.7, 202.70-202.78, 202.8, 202.80-202.88, 202.9, 202.90-202.98	C82.0, C82.00-C82.09, C82.1, C82.10-C82.19, C82.2, C82.20-C82.29, C82.3, C82.30-C82.39, C82.4, C82.40-C82.49, C82.5, C82.50-C82.59, C82.6, C82.60-C82.69, C82.8, C82.80-C82.89, C82.9, C82.90-C82.99, C83.0, C83.00-C83.09, C83.1, C83.10-C83.19, C83.3, C83.30-C83.39, C83.5, C83.50-C83.59, C83.7, C83.70-C83.79, C83.8, C83.80-C83.89, C83.9, C83.90-C83.99, C84.0, C84.00-C84.09, C84.1, C84.10-C84.19, C84.4, C84.40-C84.49, C84.6, C84.60-C84.69, C84.7, C84.70-C84.79, C84.9, C84.90-C84.99, C84.A, C84.A0-C84.A9, C84.Z, C84.Z0-C84.Z9, C85.1, C85.10-C85.19, C85.2, C85.20-C85.29, C85.8, C85.80-C85.89, C85.9, C85.90-C85.99, C86.0-C86.6, C96.2, C96.20-C96.22, C96.29, C96.4, C96.0, C96.A, C96.Z
Ovarian cancer	183, 183.0, 236.2	C56, C56.1 C56.2, C56.9, D39.10

ICD-9-CM/ICD-10-CM International Classification of Diseases-9th/10th Revision Clinical Modification

Online Resource 5 Description of methodology used to identify chemotherapy regimens and cycles in the Optum™ database

Chemotherapy course

A chemotherapy course was defined as sequential administration of chemotherapy agents separated by a less than 60-day gap. The start of the chemotherapy course was the date of initial chemotherapy administration (i.e., index date). The end date was the date of last administration of chemotherapy with a subsequent gap of 60 days or more. The first eligible chemotherapy course during the study period was considered the relevant episode of interest.

Chemotherapy cycles

Chemotherapy cycles were identified within the first eligible chemotherapy course during the study period. The first cycle of the course began with the index date and ended with the first service date for the next administration of chemotherapy occurring ≥6 days—but no more than 60 days—after the index date. If a second chemotherapy cycle did not commence prior to day 60, if there was an unplanned change in the chemotherapy regimen (i.e., based on expert opinion regarding agents received in the first cycle versus subsequent cycles), or if radiation therapy was initiated, both the first cycle of chemotherapy and the course of chemotherapy were considered to have been completed 35 days following the beginning of the cycle or on the date of change in treatment, as appropriate. The second and all subsequent cycles of chemotherapy during the index course were similarly defined.

Chemotherapy agents/regimens

Chemotherapy regimens were determined based on a review of all Healthcare Common Procedure Coding System (HCPCS) Level II codes for parenterally administered antineoplastic agents on claims with service dates from day 1 of chemotherapy administration through day 6 of each cycle of chemotherapy. Regimens were categorized based on the specific combination of agents during the course as well as periodicity (i.e., weekly, bi-weekly, tri-weekly, monthly, based on the observed duration of cycle 1), as feasible. Allowable periodicity was identified for each cycle using oncologist guidance to allow for shorter or longer than usual cycles: e.g., a 3-weekly fluorouracil plus epirubicin and cyclophosphamide (FEC) regimen or docetaxel plus cyclophosphamide (TC) regimen could occur at 18- to 31-day intervals instead of a fixed 21-day interval. Chemotherapy regimens were designated as “high” (>20%) or “intermediate” (10-20%) in terms of FN risk as defined by the NCCN® clinical practice guidelines [1].

Reference

1. Crawford J, Becker PS, Armitage JO, Blayney DW, Chavez J, Curtin P, Dinner S, Fynan T, Gojo I, Griffiths EA, Hough S, Kloth DD, Kuter DJ, Lyman GH, Mably M, Mukherjee S, Patel S, Perez LE, Poust A, Rampal R, Roy V, Rugo HS, Saad AA, Schwartzberg LS, Shayani S, Talbott M, Vadhan-Raj S, Vasu S, Wadleigh M, Westervelt P, Burns JL, Pluchino L (2017) Myeloid Growth Factors, Version 2.2017, NCCN Clinical Practice Guidelines in Oncology. J Natl Compr Canc Netw 15:1520-1541

Online Resource 6 List of codes use to identify sG-CSF

sG-CSF originator	Code	Code type	sG-CSF biosimilar	Code	Code type
Filgrastim	J1440	HCPCS	Filgrastim-sndz	Q5101	HCPCS
Filgrastim	J1441	HCPCS	Filgrastim-sndz	61314031810	NDC
Filgrastim	J1442	HCPCS	Filgrastim-sndz	61314032601	NDC
Filgrastim	54569482400	NDC	Filgrastim-sndz	61314032610	NDC
Filgrastim	54868252200	NDC	Filgrastim-sndz	61314031801	NDC
Filgrastim	54868252201	NDC	Filgrastim-sndz	61314030401	NDC
			Figrastim-sndz	61314030410	NDC
Filgrastim	54868305000	NDC	Filgrastim-sndz	61314031210	NDC
Filgrastim	54868502000	NDC	Filgrastim-sndz	61314031201	NDC
Filgrastim	55513020901	NDC	Tbo-filgrastim	J1446	HCPCS
Filgrastim	55513020910	NDC	Tbo-filgrastim	J1447	HCPCS
Filgrastim	55513020991	NDC	Tbo-filgrastim	6345991001	NDC
Filgrastim	55513034701	NDC	Tbo-filgrastim	6345991015	NDC
Filgrastim	55513034710	NDC	Tbo-filgrastim	6345991011	NDC
Filgrastim	55513034801	NDC	Tbo-filgrastim	6345991017	NDC
Filgrastim	55513034810	NDC	Tbo-filgrastim	6345991036	NDC
Filgrastim	55513053001	NDC	Tbo-filgrastim	6345991217	NDC
Filgrastim	55513053010	NDC	Tbo-filgrastim	6345991218	NDC
Filgrastim	55513054601	NDC	Tbo-filgrastim	6345991236	NDC
Filgrastim	55513054610	NDC	Tbo-filgrastim	6345991212	NDC
Filgrastim	55513092401	NDC	Tbo-filgrastim	6345991215	NDC
Filgrastim	55513092410	NDC	Tbo-filgrastim	6345991211	NDC
Filgrastim	55513092491	NDC	Tbo-filgrastim	6345991201	NDC
			Tbo-filgrastim	6345991018	NDC
			Tbo-filgrastim	6345991012	NDC

HCPCS Healthcare Common Procedure Coding System, *NDC* National Drug Code, *sG-CSF* short-acting granulocyte colony-stimulating factor

Online Resource 7 Algorithm used to identify pegfilgrastim by mode of delivery

Pegfilgrastim categories	Code	Code type	Period
Pegfilgrastim PFS	54868-5229-00	NDC	01/01/2012 to 12/31/2017
Pegfilgrastim PFS	55513-0190-01	NDC	01/01/2012 to 12/31/2017
Pegfilgrastim PFS	J2505	HCPCS	01/01/2012 to 03/01/2015
Pegfilgrastim OBI	55513-0192-01	NDC	01/01/2012 to 12/31/2017
Pegfilgrastim OBI	J2505 and CPT 96377[a] on the same day	HCPCS, CPT	01/01/2017 to 12/31/2017
Pegfilgrastim route unknown[b]	J2505	HCPCS	03/01/2015 to 12/31/2017

[a]CPT 96377: Application of OBI (includes cannula insertion) for timed subcutaneous injection
[b]Pegfilgrastim route unknown: pegfilgrastim users who could not be classified as pegfilgrastim PFS or OBI
CPT Current Procedural Terminology, *HCPCS* Healthcare Common Procedure Coding System, *NDC* National Drug Code, *PFS* prefilled syringe, *OBI* on-body injector

Online Resource 8 Characteristics and comorbidities of patients receiving prophylactic G-CSF in the first cycle, stratified by cancer type and by high/intermediate FN risk of the chemotherapy regimen

Characteristics	Overall		Breast cancer		Lung cancer		Ovarian cancer		Colorectal cancer		NHL	
	No G-CSF	G-CSF	No G-CSF	G-CSF	No G-CSF	G-CSF	No G-CSF	G-CSF	No G-CSF	G-CSF	No G-CSF	G-CSF
High/intermediate FN risk	*N* = 22,868		*N* = 11,513		*N* = 4273		*N* = 1287		*N* = 3765		*N* = 2030	
Number of patients	12,379 (54.1)	10,489 (45.9)	4585 (39.8)	6928 (60.2)	2845 (66.6)	1428 (33.4)	1016 (78.9)	271 (21.1)	3434 (91.2)	331 (8.8)	499 (24.6)	1531 (75.4)
Patients receiving chemotherapy												
High FN risk	2010 (23.9)	6414 (76.1)	1949 (23.6)	6321 (76.4)	3 (75.0)	1 (25.0)	31 (86.1)	5 (13.9)	0 (0)	0 (0)	27 (23.7)	87 (76.3)
Intermediate FN risk	10,369 (71.8)	4075 (28.2)	2636 (81.3)	607 (18.7)	2842 (66.6)	1427 (33.4)	985 (78.7)	266 (21.3)	3434 (91.2)	331 (8.8)	472 (24.6)	1444 (75.4)
Intermediate FN risk and ≥1 risk factor[a]	6853 (73.6)	2456 (26.4)	1359 (83.0)	279 (17.0)	2105 (66.4)	1064 (33.6)	617 (78.7)	167 (21.3)	2590 (91.3)	248 (8.7)	182 (20.7)	698 (79.3)
Mean age, years (SD)	64.4 (12.2)	62.2 (12.4)	62.0 (12.7)	59.0 (12.0)	70.2 (8.5)	70.0 (8.1)	65.0 (12.7)	66.6 (11.1)	63.2 (12.0)	63.5 (12.1)	61.6 (13.5)	68.1 (12.1)
Mean age category, years (SD)												
18–44 years	840 (6.8)	1051 (10.0)	472 (10.3)	937 (13.5)	5 (0.2)	5 (0.4)	60 (5.9)	11 (4.1)	249 (7.3)	26 (7.9)	54 (10.8)	72 (4.7)
45–54 years	1870 (15.1)	1846 (17.6)	874 (19.1)	1579 (22.8)	134 (4.7)	53(3.7)	145 (14.3)	31 (11.4)	634 (18.5)	54 (16.3)	83 (16.6)	129 (8.4)
55–64 years	2743 (22.2)	2502 (23.9)	1076 (23.5)	1787 (25.8)	544 (19.1)	284 (19.9)	241 (23.7)	63 (23.2)	752 (21.9)	80 (24.2)	130 (26.1)	288 (18.8)
65–74 years	4282 (34.6)	3439 (32.8)	1411 (30.8)	2028 (29.3)	1217 (42.8)	655 (45.9)	315 (31.0)	96 (35.4)	1198 (34.9)	106 (32.0)	141 (28.3)	554 (36.2)
≥75 years	2644 (21.4)	1651 (15.7)	752 (16.4)	597 (8.6)	945 (33.2)	431 (30.2)	255 (25.1)	70 (25.8)	601 (17.5)	65 (19.6)	91 (18.2)	488 (31.9)
Sex, female	8722 (70.5)	8778 (83.7)	4585 (100.0)	6928 (100.0)	1347 (47.3)	700 (49.0)	1016 (100.0)	271 (100.0)	1580 (46.0)	154 (46.5)	194 (38.9)	725 (47.4)
Number of completed cycles: mean (SD) [median]	3.2 (2.5) [2]	4.0 (2.10) [4]	2.7 (2.2) [1]	4.2 (2.1) [4]	2.0 (1.6) [1]	3.0 (1.9) [3]	2.4 (1.9) [1)	3.9 (2.0) [4]	5.0 (2.7) [5]	5.1 (2.7) [3]	3.5 (2.1) [3]	3.8 (2.0) [4]
Comorbidities, *n* (%)												
Cardiovascular disease[b]	1961 (15.8)	1141 (10.9)	352 (7.7)	354 (5.1)	841 (29.6)	445 (31.2)	134 (13.2)	48 (17.7)	565 (16.5)	63 (19.0)	69 (13.8)	231 (15.1)
Stroke	509 (4.1	306 (2.9)	98 (2.1)	99 (1.4)	265 (9.3)	138 (9.7)	18 (1.8)	8 (3.0)	113 (3.3)	9 (2.7)	15 (3.0)	52 (3.4)
PVD	904 (7.3)	481 (4.6)	152 (3.3)	134 (1.9)	357 (12.5)	179 (12.5)	84 (8.3)	28 (10.3)	283 (8.2)	38 (11.5)	28 (5.6)	102 (6.7)
MI	407 (3.3)	211 (2.0)	49 (1.1)	57 (0.8)	194 (6.8)	98 (6.9)	19 (1.9)	4 (1.5)	131 (3.8)	10 (3.0)	14 (2.8)	42 (2.7)
HF	704 (5.7)	410 (3.9)	142 (3.1)	131 (1.9)	316 (11.1)	169 (11.8)	41 (4.0)	16 (5.9)	184 (5.4)	19 (5.7)	21 (4.2)	75 (4.9)
Infection	2724 (22.0)	1676 (16.0)	492 (10.7)	661 (9.5)	1075 (37.8)	535 (37.5)	201 (19.8)	62 (22.9)	871 (25.4)	98 (29.6)	85 (17.0)	320 (20.9)
Metastasis	3839 (31.0)	1595 (15.2)	859 (18.7)	888 (12.8	704 (24.7)	369 (25.8)	421 (41.4)	106 (39.1)	1835 (53.4)	171 (51.7)	20 (4.0)	61 (4.0)

Characteristics	Overall		Breast cancer		Lung cancer		Ovarian cancer		Colorectal cancer		NHL	
	No G-CSF	G-CSF	No G-CSF	G-CSF	No G-CSF	G-CSF	No G-CSF	G-CSF	No G-CSF	G-CSF	No G-CSF	G-CSF
Type 2 diabetes	2610 (21.1)	1926 (18.4)	837 (18.3)	1070 (15.4)	702 (24.7)	379 (26.5)	158 (15.6)	43 (15.9)	818 (23.8)	88 (26.6)	95 (19.0)	346 (22.6)
CKD	934 (7.5)	581 (5.5)	244 (5.3)	228 (3.3)	300 (10.5)	165 (11.6)	65 (6.4)	27 (10.0)	278 (8.1)	20 (6.0)	47 (9.4)	141 (9.2)
Liver disease	558 (4.5)	251 (2.4)	87 (1.9)	85 (1.2)	107 (3.8)	55 (3.9)	46 (4.5)	15 (5.5)	303 (8.8)	33 (10.0)	15 (3.0)	63 (4.1)
COPD	1010 (8.2)	560 (5.3)	141 (3.1)	127 (1.8)	625 (22.0)	324 (22.7)	34 (3.3)	15 (5.5)	192 (5.6)	18 (5.4)	18 (3.6)	76 (5.0)
HIV/AIDS	21 (0.2)	14 (0.1)	0 (0)	4 (0.1)	5 (0.2)	3 (0.2)	1 (0.1)	0 (0)	10 (0.3)	0 (0)	5 (1.0)	7 (0.5)
Organ transplantation	24 (0.2)	19 (0.2)	4 (0.1)	7 (0.1)	10 (0.4)	5 (0.4)	1 (0.1)	0 (0)	4 (0.1)	0 (0)	5 (1.0)	7 (0.5)
Neutropenia hospitalization	47 (0.4)	61 (0.6)	10 (0.2)	12 (0.2)	13 (0.5)	10 (0.7)	4 (0.4)	1 (0.4)	5 (0.1)	0 (0)	15 (3.0)	38 (2.5)
Charlson comorbidity index, mean (SD)	7.2 (3.7)	5.7 (3.6)	5.8 (3.7)	5.1 (3.4)	8.4 (3.4)	8.9 (3.3)	7.8 (3.3)	8.0 (3.3)	8.4 (3.3)	8.4 (3.5)	4.2 (3.0)	4.4 (3.0)
High FN risk	*N* = 8424		*N* = 8270		*N* = 4		*N* = 36		*N* = 0		*N* = 114	
Number of patients	2010 (23.9)	6414 (76.1)	1949 (23.6)	6321 (76.4)	3 (75.0)	1 (25.0)	31 (86.1)	5 (13.9)	0 (0)	0 (0)	27 (23.7	87 (76.3)
Mean age, years (SD)	59.3 (11.8)	58.8 (12.0)	59.2 (11.7)	58.8 (12.0)	75.3 (4.7)	76.0 (N/A)	66.3 (12.3)	72.0 (11.1)	0 (0)	0 (0)	55.9 (15.6)	60.3 (13.8)
Mean age category, years (SD)												
18–44 years	236 (11.7)	894 (13.9)	229 (11.7)	882 (14.0)	0 (0)	0 (0)	1 (3.2)	0 (0)	0 (0)	0 (0)	6 (22.2)	12 (13.8)
45–54 years	486 (24.2)	1472 (22.9)	475 (24.4)	1459 (23.1)	0 (0)	0 (0)	5 (16.1)	0 (0)	0 (0)	0 (0)	6 (22.2)	13 (14.9)
55–64 years	521 (25.9)	1684 (26.3)	508 (26.1)	1655 (26.2)	0 (0)	0 (0)	8 (25.8)	2 (40.0)	0 (0)	0 (0)	5 (18.5)	27 (31.0)
65–74 years	593 (29.5)	1831 (28.5)	579 (29.7)	1808 (28.6)	1 (33.3)	0 (0)	7 (22.6)	1 (20.0)	0 (0)	0 (0)	6 (22.2)	22 (25.3)
≥75 years	174 (8.7)	533 (8.3)	158 (8.1)	517 (8.2)	2 (66.7)	1 (100.0)	10 (32.3)	2 (40.0)	0 (0)	0 (0)	4 (14.8)	13 (14.9)
Sex, female	1987 (98.9)	6359 (99.1)	1949(100.0)	6321 (100.0)	2 (66.7)	0 (0)	31 (100.0)	5 (100.0)	0 (0)	0 (0)	5 (18.5)	33 (37.9)
Number of completed cycles, mean (SD), [median]	3.2 (1.9) [4]	4.1 (2.0) [4]	3.3 (1.9) [4]	4.2 (2.0) [4]	1.0 (0) [1]	1 (N/A) [1]	1.5 (1.4) [1]	2.6 (3.1) [1]	N/A	N/A	2.8 (1.8) [2]	3.1 (1.8) [3]
Comorbidities												
Cardiovascular disease[b]	104 (5.2)	325 (5.1)	90 (4.6)	309 (4.9)	0 (0)	0 (0)	7 (22.6)	1 (20.0)	0 (0)	0 (0)	7 (25.9)	15 (17.2)
Stroke	31 (1.5)	92 (1.4)	28 (1.4)	90 (1.4)	0 (0)	0 (0)	1 (3.2)	0 (0)	0 (0)	0 (0)	2 (7.4)	2 (2.3)
PVD	41 (2.0)	121 (1.9)	36 (1.8)	111 (1.8)	0 (0)	0 (0)	3 (9.7)	1 (20.0)	0 (0)	0 (0)	2 (7.4)	9 (10.3)
MI	20 (1.0)	61 (1.0)	16 (0.8)	55 (0.9)	0 (0)	0 (0)	3 (9.7)	0 (0)	0 (0)	0 (0)	1 (3.7)	6 (6.9)
HF	39 (1.9)	121 (1.9)	36 (1.8)	116 (1.8)	0 (0)	0 (0)	1 (3.2)	0 (0)	0 (0)	0 (0)	2 (7.4)	5 (5.7)
Infection	202 (10.0)	608 (9.5)	185 (9.5)	588 (9.3)	2 (66.7)	0 (0)	10 (32.3)	0 (0)	0 (0)	0 (0)	5 (18.5)	20 (23.0)

Characteristics	Overall		Breast cancer		Lung cancer		Ovarian cancer		Colorectal cancer		NHL	
	No G-CSF	G-CSF	No G-CSF	G-CSF	No G-CSF	G-CSF	No G-CSF	G-CSF	No G-CSF	G-CSF	No G-CSF	G-CSF
Metastasis	206 (10.2)	770 (12.0)	185 (9.5)	764 (12.1)	0 (0)	0 (0)	19 (61.3)	2 (40.0)	0 (0)	0 (0)	2 (7.4)	4 (4.6)
Type 2 diabetes	323 (16.1)	968 (15.1)	310 (15.9)	949 (15.0)	0 (0)	0 (0)	7 (22.6)	0 (0)	0 (0)	0 (0)	6 (22.2)	19 (21.8)
CKD	78 (3.9)	214 (3.3)	73 (3.7)	205 (3.2)	0 (0)	0 (0)	2 (6.5)	1 (20.0)	0 (0)	0 (0)	3 (11.1)	8 (9.2)
Liver disease	27 (1.3)	76 (1.2)	25 (1.3)	70 (1.1)	0 (0)	0 (0)	1 (3.2)	0 (0)	0 (0)	0 (0)	1 (3.7)	6 (6.9)
COPD	35 (1.7)	114 (1.8)	33 (1.7)	109 (1.7)	1 (33.3)	0 (0)	0 (0)	2 (40.0)	0 (0)	0 (0)	1 (3.7)	3 (3.4)
HIV/AIDS	2 (0.1)	4 (0.1)	0 (0)	2 (0)	0 (0)	0 (0)	0 (0)	0 (0)	0 (0)	0 (0)	2 (7.4)	2 (2.3)
Organ transplantation	1 (0)	7 (0.1)	0 (0)	7 (0.1)	0 (0)	0 (0)	0 (0)	0 (0)	0 (0)	0 (0)	1 (3.7)	0 (0)
Neutropenia hospitalization	5 (0.2)	9 (0.1)	3 (0.2)	7 (0.1)	0 (0)	0 (0)	0 (0)	0 (0)	0 (0)	0 (0)	2 (7.4)	2 (2.3)
Charlson comorbidity index, mean (SD)	4.7 (3.3)	5.0 (3.3)	4.6 (3.3)	5 (3.3)	7.0 (4.4)	2.0 (N/A)	9.6 (2.1)	10.6 (2.1)	N/A	N/A	4.2 (2.9)	4.9 (3.3)
Intermediate FN risk	*N* = 14,444		*N* = 3243		*N* = 4269		*N* = 1251		*N* = 3765		*N* = 1916	
Number of patients	10,369 (71.8)	4075 (28.2)	2636 (81.3)	607 (18.7)	2842 (66.6)	1427 (33.4)	985 (78.7)	266 (21.3)	3434 (91.2)	331 (8.8)	472 (24.6)	1444 (75.4)
Mean age, years (SD)	65.4 (12.0)	67.5 (11.1)	64.1 (13.1)	61.6 (12.1)	70.2 (8.5)	70.0 (8.1)	65 (12.7)	66.5 (11.1)	63.2 (12.0)	63.5 (12.2)	61.9 (13.3)	68.6 (11.8)
Mean age category, years (SD)												
18–44 years	604 (5.8)	157 (3.9)	243 (9.2)	55 (9.1)	5 (0.2)	5 (0.4)	59 (6.0)	11 (4.1)	249 (7.3)	26 (7.9)	48 (10.2)	60 (4.2)
45–54 years	1384 (13.3)	374 (9.2)	399 (15.1)	120 (19.8)	134 (4.7)	53 (3.7)	140 (14.2)	31 (11.7)	634 (18.5)	54 (16.3)	77 (16.3)	116 (8.0)
55–64 years	2222 (21.4)	818 (20.1)	568 (21.5)	132 (21.7)	544 (19.1)	284 (19.9)	233 (23.7)	61 (22.9)	752 (21.9)	80 (24.2)	125 (26.5)	261 (18.1)
65–74 years	3689 (35.6)	1608 (39.5)	832 (31.6)	220 (36.2)	1216 (42.8)	655 (45.9)	308 (31.3)	95 35.7)	1198 (34.9)	106 (32.0)	135 (28.6)	532 (36.8)
≥75 years	2470 (23.8)	1118 (27.4)	594 (22.5)	80 (13.2)	943 (33.2)	430 (30.1)	245 (24.9)	68 (25.6)	601 (17.5)	65 (19.6)	87 (18.4)	475 (32.9)
Sex, female	6735 (65.0)	2419 (59.4)	2636 (100.0)	607 (100.0)	1345 (47.3)	700 (49.1)	985 (100.0)	266 (100.0)	1580 (46.0)	154 (46.5)	189 (40.0)	692 (47.9)
Number of completed cycles, mean (SD), [median]	3.2 (2.6) [2]	3.8 (2.3) [4]	2.2 (2.2) [1]	4.9 (2.6) [5]	2.0 (1.6) [1]	3.0 (1.9) [3]	2.4 (1.9) [1]	3.9 (1.9) [4]	5.0 (2.7) [5]	5.1 (2.7) [5]	3.6 (2.1) [3.0]	3.8 (2.0) [4.0]
Comorbidities												
Cardiovascular disease[b]	1857 (17.9)	816 (20.0)	262 (9.9)	45 (7.4)	841 (29.6)	445 (31.2)	127 (12.9)	47 (17.7)	565 (16.5)	63 (19.0)	62 (13.1)	216 (15.0)
Stroke	478 (4.6)	214 (5.3)	70 (2.7)	9 (1.5)	265 (9.3)	138 (9.7)	17 (1.7)	8 (3.0)	113 (3.3)	9 (2.7)	13 (2.8)	50 (3.5)
PVD	863 (8.3)	360 (8.8)	116 (4.4)	23 (3.8)	357 (12.6)	179 (12.5)	81 (8.2)	27 (10.2)	283 (8.2)	38 (11.5)	26 (5.5)	93 (6.4)
MI	387 (3.7)	150 (3.7)	33 (1.3)	2 (0.3)	194 (6.8)	98 (6.9)	16 (1.6)	4 (1.5)	131 (3.8)	10 (3.0)	13 (2.8)	36 (2.5)
HF	665 (6.4)	289 (7.1)	106 (4.0)	15 (2.5)	316 (11.1)	169 (11.8)	40 (4.1)	16 (6.0)	184 (5.4)	19 (5.7)	19 (14.0)	70 (4.8)

Characteristics	Overall		Breast cancer		Lung cancer		Ovarian cancer		Colorectal cancer		NHL	
	No G-CSF	G-CSF	No G-CSF	G-CSF	No G-CSF	G-CSF	No G-CSF	G-CSF	No G-CSF	G-CSF	No G-CSF	G-CSF
Infection	2522 (24.3)	1068 (26.2)	307 (11.6)	73 (12.0)	1073 (37.8)	535 (37.5)	191 (19.4)	62 (23.3)	871 (25.4)	98 (29.6)	80 (16.9)	300 (20.8)
Metastasis	3633 (35.0)	825 (20.2)	674 (25.6)	124 (20.4)	704 (24.8)	369 (25.9)	402 (40.8)	104 (39.1)	1835 (53.4)	171 (51.7)	18 (3.8)	57 (3.9)
Type 2 diabetes	2287 (22.1)	958 (23.5)	527 (20.0)	121 (19.9)	702 (24.7)	379 (26.6)	151 (15.3)	43 (16.2)	818 (23.8)	88 (26.6)	89 (18.9)	327 (22.6)
CKD	856 (8.3)	367 (9.0)	171 (6.5)	23 (3.8)	300 (10.6)	165 (11.6)	63 (6.4)	26 (9.8)	278 (8.1)	20 (6.0)	44 (9.3)	133 (9.2)
Liver disease	531 (5.1)	175 (4.3)	62 (2.4)	15 (2.5)	107 (3.8)	55 (3.9)	45 (4.6)	15 (5.6)	303 (8.8)	33 (10.0)	14 (3.0)	57 (3.9)
COPD	975 (9.4)	446 (10.9)	108 (4.1)	18 (3.0)	624 (22.0)	324 (22.7)	34 (3.5)	13 (4.9)	192 (5.6)	18 (5.4)	17 (3.6)	73 (5.1)
HIV/AIDS	19 (0.2)	10 (0.2)	0 (0)	2 (0.3)	5 (0.2)	3 (0.2)	1 (0.1)	0 (0)	10 (0.3)	0 (0)	3 (0.6)	5 (0.3)
Organ transplantation	23 (0.2)	12 (0.3)	4 (0.2)	0 (0)	10 (0.4)	5 (0.4)	1 (0.1)	0 (0)	4 (0.1)	0 (0)	4 (0.8)	7 (0.5)
Neutropenia hospitalization	42 (0.4)	52 (1.3)	7 (0.3)	5 (0.8)	13 (0.5)	10 (0.7)	4 (0.4)	1 (0.4)	5 (0.1)	0 (0)	13 (2.8)	36 (2.5)
Charlson comorbidity index, mean (SD)	7.7 (3.6)	6.8 (3.8)	6.7 (3.8)	6.5 (3.6)	8.4 (3.4)	8.9 (3.2)	7.7 (3.3)	7.9 (3.3)	8.4 (3.3)	8.4 (3.5)	4.2 (3.0)	4.4 (3.0)
Intermediate FN risk with ≥1 risk factor[a]	*N* = 9309		*N* = 1638		*N* = 3169		*N* = 784		*N* = 2838		*N* = 880	
Number of patients	6853 (73.6)	2456 (26.4)	1359 (83.0)	279 (17.0)	2105 (66.4)	1064 (33.6)	617 (78.7)	167 (21.3)	2590 (91.3)	248 (8.7)	182 (20.7)	698 (79.3)
Mean age, years (SD)	67.5 (11.0)	69.4 (9.7)	67.8 (11.7)	64.9 (10.8)	71.2 (8.0)	70.6 (7.9)	66.8 (11.8)	67.5 (11.0)	64.6 (11.5)	65.0 (11.8)	67.6 (12.2)	71.6 (9.3)
Mean age category, years (SD)												
18–44 years	249 (3.6)	45 (1.8)	65 (4.8)	11 (3.9)	2 (0.1)	1 (0.1)	23 (3.7)	7 (4.2)	148 (5.7)	16 (6.5)	11 (6.0)	10 (1.4)
45–54 years	695 (10.1)	152 (6.2)	135 (9.9)	40 (14.3)	67 (3.2)	38 (3.6)	78 (12.6)	14 (8.4)	405 (15.6)	33 (13.3)	10 (5.5)	27 (3.9)
55–64 years	1287 (18.8)	436 (17.8)	254 (18.7)	58 (20.8)	339 (16.1)	186 (17.5)	121 (19.6)	37 (22.2)	540 (20.8)	57 (23.0)	33 (18.1)	98 (14.0)
65–74 years	2707 (39.5)	1060 (43.2)	496 (36.5)	121 (43.4)	924 (43.9)	502 (47.2)	226 (36.6)	57 (34.1)	989 (38.2)	85 (34.3)	72 (39.6)	295 (42.3)
≥75 years	1915 (27.9)	763 (31.1)	409 (30.1)	49 (17.6)	773 (36.7)	337 (31.7)	169 (27.4)	52 (31.1)	508 (19.6)	57 (23.0)	56 (30.8)	268 (38.4)
Sex, female	4224 (61.6)	1406 (57.2)	1359 (100.0)	279 (100.0)	987 (46.9)	506 (47.6)	617 (100.0)	167 (100.0)	1184 (45.7)	114 (46.0)	77 (42.3)	340 (48.7)
Number of completed cycles, mean (SD), [median]	3.2 (2.6) [2]	3.7 (2.2) [4]	2.1 (2.1) [1]	4.8 (2.5) [5]	2.0 (1.6) [1]	3.1 (1.9) [3]	2.3 (1.9) [1]	3.9 (2.0) [4.0]	5.1 (2.7) [6.0]	5.1 (2.6) [5.0]	3.4 (2.1) [3]	3.7 (2.0) [4.0]
Comorbidities												
Cardiovascular disease[b]	1857 (27.1)	816 (33.2)	262 (19.3)	45 (16.1)	841 (40.0)	445 (41.8)	127 (20.6)	47 (28.1)	565 (21.8)	63 (25.4)	62 (34.1)	216 (30.9)
Stroke	478 (7.0)	214 (8.7)	70 (5.2)	9 (3.2)	265 (12.6)	138 (13.0)	17 (2.8)	8 (4.8)	113 (4.4)	9 (3.6)	13 (7.1)	50 (7.2)
PVD	863 (12.6)	360 (14.7)	116 (8.5)	23 (8.2)	357 (17.0)	179 (16.8)	81 (13.1)	27 (16.2)	283 (10.9)	38 (15.3)	26 (14.3)	93 (13.3)
MI	387 (5.6)	150 (6.1)	33 (2.4)	2 (0.7)	194 (9.2)	98 (9.2)	16 (2.6)	4 (2.4)	131 (5.1)	10 (4.0)	13 (7.1)	36 (5.2)

Characteristics	Overall		Breast cancer		Lung cancer		Ovarian cancer		Colorectal cancer		NHL	
	No G-CSF	G-CSF	No G-CSF	G-CSF	No G-CSF	G-CSF	No G-CSF	G-CSF	No G-CSF	G-CSF	No G-CSF	G-CSF
HF	665 (9.7)	289 (11.8)	106 (7.8)	15 (5.4)	316 (15.0)	169 (15.9)	40 (6.5)	16 (9.6)	184 (7.1)	19 (7.7)	19 (10.4)	70 (10.0)
Infection	2254 (32.9)	961 (39.1)	247 (18.2)	60 (21.5)	1002 (47.6)	508 (47.7)	158 (25.6)	52 (31.1)	787 (30.4)	88 (35.5)	60 (33.0)	253 (36.2)
Metastasis	3633 (53.0)	825 (33.6)	674 (49.6)	124 (44.4)	704 (33.4)	369 (34.7)	402 (65.2)	104 (62.3)	1835 (70.8)	171 (69.0)	18 (9.9)	57 (8.2)
Type 2 diabetes	2287 (33.4)	958 (39.0)	527 (38.8)	121 (43.4)	702 (33.3)	379 (35.6)	151 (24.5)	43 (25.7)	818 (31.6)	88 (35.5)	89 (48.9)	327 (46.8)
CKD	856 (12.5)	367 (14.9)	171 (12.6)	23 (8.2)	300 (14.3)	165 (15.5)	63 (10.2)	26 (15.6)	278 (10.7)	20 (8.1)	44 (24.2)	133 (19.1)
Liver disease	531 (7.7)	175 (7.1)	62 (4.6)	15 (5.4)	107 (5.1)	55 (5.2)	45 (7.3)	15 (9.0)	303 (11.7)	33 (13.3)	14 (7.7)	57 (8.2)
COPD	975 (14.2)	446 (18.2)	108 (7.9)	18 (6.5)	624 (29.6)	324 (30.5)	34 (5.5)	13 (7.8)	192 (7.4)	18 (7.3)	17 (9.3)	73 (10.5)
HIV/AIDS	18 (0.3)	9 (0.4)	0 (0)	1 (0.4)	5 (0.2)	3 (0.3)	1 (0.2)	0 (0)	9 (0.3)	0 (0)	3 (1.6)	5 (0.7)
Organ transplantation	23 (0.3)	12 (0.5)	4 (0.3)	0 (0)	10 (0.5)	5 (0.5)	1 (0.2)	0 (0)	4 (0.2)	0 (0)	4 (2.2)	7 (1.0)
Neutropenia hospitalization	37 (0.5)	49 (2.0)	5 (0.4)	5 (1.8)	13 (0.6)	10 (0.9)	3 (0.5)	1 (0.6)	4 (0.2)	0 (0)	12 (6.6)	33 (4.7)
Charlson comorbidity index, mean (SD)	8.7 (3.2)	8.0 (3.6)	8.3 (3.4)	8.2 (3.3)	8.8 (3.4)	9.3 (3.2)	8.8 (2.7)	8.7 (3.0)	9.0 (2.8)	9.2 (3.0)	5.6 (3.3)	5.3 (3.2)

Data presented as *n* (%) unless otherwise stated

Note: Column percentages are used except for row percentages for number of patients and FN risk of the chemotherapy regimen (high, intermediate, or intermediate with ≥1 patient-level risk factors)

[a]Risk factors include age >65 years, metastatic disease, diabetes mellitus, cardiovascular disease, COPD, liver or renal dysfunction, and HIV/AIDS

[b]Cardiovascular disease includes MI, HF, PVD, or stroke

CKD chronic kidney disease, *COPD* chronic obstructive pulmonary disease, *HF* heart failure, *HIV/AIDS* human immunodeficiency virus infection and acquired immune deficiency syndrome, *MI* myocardial infarction, *N/A* not applicable, *NHL* non-Hodgkin lymphoma, *prophylactic G-CSF* primary prophylaxis with granulocyte colony-stimulating factor, *PVD* peripheral vascular disease, *sG-CSF* short-acting granulocyte colony-stimulating factor, *SD* standard deviation

Online Resource 9 Characteristics and comorbidities of patients receiving prophylactic G-CSF in the first cycle, stratified by G-CSF type and by high/intermediate FN risk of the chemotherapy regimen

Characteristics	Any G-CSF		Any pegfilgrastim				Any sG-CSF		
	Any pegfilgrastim	Any sG-CSF	PFS	PFS ideal time (days 1–3)	OBI	Route unknown[a]	Filgrastim	Filgrastim-sndz	Tbo-filgrastim
High/intermediate FN risk		*N* = 10,489			*N* = 10,146			*N* = 343	
Number of patients	10,146 (96.7)	343 (3.3)	5697 (56.2)	4884 (85.7)	1383 (13.6)	3066 (30.2)	232 (67.6)	55 (16.0)	56 (16.3)
Patients receiving chemotherapy									
High FN risk	6288 (98.0)	126 (2.0)	3536 (56.2)	2999 (84.8)	909 (14.5)	1843 (29.3)	86 (68.3)	17 (13.5)	23 (18.3)
Intermediate FN risk	3858 (94.7)	217 (5.3)	2161 (56.0)	1885 (87.2)	474 (12.3)	1223 (31.7)	146 (67.3)	38 (17.5)	33 (15.2)
Intermediate FN risk and ≥1 risk factor[b]	2319 (94.4)	137 (5.6)	1258 (54.2)	1114 (88.6)	305 (13.2)	756 (32.6)	92 (67.2)	29 (21.2)	16 (11.7)
Mean age, years (SD)	62.1 (12.4)	65.2 (11.4)	61.2 (12.4)	61.9 (12.3)	61.8 (12.5)	63.8 (12.4)	64.6 (11.8)	68.3 (10.1)	64.9 (10.7)
Mean age category, years (SD)									
18–44 years	1028 (10.1)	23 (6.7)	618 (10.8)	504 (10.3)	146 (10.6)	264 (8.6)	18 (7.8)	1 (1.8)	4 (7.1)
45–54 years	1806 (17.8)	40 (11.7)	1113 (19.5)	892 (18.3)	248 (17.9)	445 (14.5)	30 (12.9)	6 (10.9)	4 (7.1)
55–64 years	2424 (23.9)	78 (22.7)	1437 (25.2)	1174 24.0)	344 (24.9)	643 (21.0)	51 (22.0)	9 (16.4)	18 (32.1)
65–74 years	3308 (32.6)	131 (38.2)	1719 (30.2)	1566 (32.1)	437 (31.6)	1152 (37.6)	85 (36.6)	28 (50.9)	18 (32.1)
≥75 years	1580 (15.6)	71 (20.7)	810 (14.2)	748 (15.3)	208 (15.0)	562 (18.3)	48 (20.7)	11 (20.0)	12 (21.4)
Sex, female	8507 (83.8)	271 (79.0)	4792 (84.1)	4103 (84.0)	1186 (85.8)	2529 (82.5)	181 (78.0)	45 (81.8)	45 (80.4)
Number of administrations per cycle, mean (SD)	N/A	3.3 (2.3)	N/A	N/A	N/A	N/A	3.2 (2.3)	3.0 (1.6)	4.3 (2.5)
Number of completed cycles, mean (SD)	4.0 (2.1)	3.3 (2.3)	4.1 (2.1)	4.3 (2.1)	4.3 (2.0)	3.8 (2.1)	3.2 (2.2)	4.0 (2.4)	3.4 (2.1)
Median (Q1, Q3)	4.0 (2.0, 6.0)	3.0 (1.0, 5.0)	4.0 (2.0, 6.0)	4.0 (3.0, 6.0)	4.0 (3.0, 6.0)	4.0 (2.0, 5.0)	3.0 (1.0, 5.0)	4.0 (1.0, 6.0)	3.0 (1.5, 5.0)
Comorbidities									
Cardiovascular disease[c]	1079 (10.6)	62 (18.1)	606 (10.6)	551 (11.3)	142 (10.3)	331 (10.8)	36 (15.5)	15 (27.3)	11 (19.6)
Stroke	295 (2.9)	11 (3.2)	203 (3.6)	191 (3.9)	26 (1.9)	66 (2.2)	5 (2.2)	4 (7.3)	2 (3.6)
PVD	445 (4.4)	36 (10.5)	235 (4.1)	211 (4.3)	63 (4.6)	147 (4.8)	24 (10.3)	6 (10.9)	6 (10.7)
MI	199 (2.0)	12 (3.5)	104 (1.8)	96 (2.0)	29 (2.1)	66 (2.2)	6 (2.6)	3 (5.5)	3 (5.4)
HF	394 (3.9)	16 (4.7)	207 (3.6)	190 (3.9)	64 (4.6)	123 (4.0)	9 (3.9)	5 (9.1)	2 (3.6)
Infection	1613 (15.9)	63 (18.4)	1002 (17.6)	868 (17.8)	178 (12.9)	433 (14.1)	46 (19.8)	11 (20.0)	6 (10.7)
Metastasis	1516 (14.9)	79 (23.0)	892 (15.7)	763 (15.6)	183 (13.2)	441 (14.4)	56 (24.1)	13 (23.6)	10 (17.9)
Type 2 diabetes	1859 (18.3)	67 (19.5)	1011 (17.7)	916 (18.8)	238 (17.2)	610 (19.9)	43 (18.5)	15 (27.3)	9 (16.1)
CKD	559 (5.5)	22 (6.4)	266 (4.7)	242 (5.0)	84 (6.1)	209 (6.8)	14 (6.0)	7 (12.7)	1 (1.8)
Liver disease	237 (2.3)	14 (4.1)	88 (1.5)	78 (1.6)	44 (3.2)	105 (3.4)	9 (3.9)	3 (5.5)	2 (3.6)

Characteristics	Any G-CSF		Any pegfilgrastim				Any sG-CSF		
	Any pegfilgrastim	Any sG-CSF	PFS	PFS ideal time (days 1–3)	OBI	Route unknown[a]	Filgrastim	Filgrastim-sndz	Tbo-filgrastim
COPD	529 (5.2)	31 (9.0)	107 (1.9)	95 (1.9)	155 (11.2)	267 (8.7)	14 (6.0)	15 (27.3)	2 (3.6)
HIV/AIDS	14 (0.1)	0 (0)	3 (0.1)	2 (0)	5 (0.4)	6 (0.2)	0 (0)	0 (0)	0 (0)
Organ transplantation	18 (0.2)	1 (0.3)	8 (0.1)	5 (0.1)	4 (0.3)	6 (0.2)	1 (0.4)	0 (0)	0 (0)
Neutropenia hospitalization	60 (0.6)	1 (0.3)	36 (0.6)	29 (0.6)	4 (0.3)	20 (0.7)	1 (0.4)	0 (0)	0 (0)
Charlson comorbidity index, mean (SD)	5.7 (3.6)	6.6 (3.7)	5.8 (3.7)	5.8 (3.7)	5.4 (3.5)	5.7 (3.6)	6.7 (3.7)	6.7 (3.8)	5.9 (3.6)
High FN risk only		*N* = 6414			*N* = 6288			*N* = 126	
Number of patients	6288 (98.0)	126 (2.0)	3536 (56.2)	2999 (84.8)	909 (14.5)	1843 (29.3)	86 (68.3)	17 (13.5)	23 (18.3)
Mean age, years (SD)	58.7 (12.0)	61.9 (10.4)	58.0 (11.9)	58.6 (12.1)	58.6 (12.2)	60.3 (12.0)	60.9 (11.0)	65.4 (9.4)	62.9 (8.6)
Mean age category, years (SD)									
18–45 years	884 (14.1)	10 (7.9)	527 (14.9)	432 (14.4)	132 (14.5)	225 (12.2)	9 (10.5)	0 (0)	1 (4.3)
45–54 years	1451 (23.1)	21 (16.7)	887 (25.1)	709 (23.6)	209 (23.0)	355 (19.3)	15 (17.4)	4 (23.5)	2 (8.7)
55–64 years	1646 (26.2)	38 (30.2)	961 (27.2)	788 (26.3)	238 (26.2)	447 (24.3)	27 (31.4)	3 (17.6)	8 (34.8)
65–74 years	1782 (28.3)	49 (38.9)	886 (25.1)	815 (27.2)	257 (28.3)	639 (34.7)	28 (32.6)	9 (52.9)	12 (52.2)
≥75 years	525 (8.3)	8 (6.3)	275 (7.8)	255 (8.5)	73 (8.0)	177 (9.6)	7 (8.1)	1 (5.9)	0 (0)
Sex, female	6236 (99.2)	123 (97.6)	3511 (99.3)	2980 (99.4)	905 (99.6)	1820 (98.8)	83 (96.5)	17 (100.0)	23 (100.0)
Number of completed cycles, mean (SD)	4.2 (2.0)	3.5 (1.9)	4.2 (2.0)	4.4 (1.9)	4.4 (1.8)	4.0 (2.0)	3.4 (1.9)	3.5 (2.0)	3.6 (1.6)
Median (Q1, Q3)	4.0 (3.0, 6.0)	4.0 (2.0, 5.0)	4.0 (3.0, 6.0)	4.0 (4.0, 6.0)	4.0 (4.0, 6.0)	4.0 (2.0, 5.0)	4.0 (1.0, 5.0)	4.0 (2.0, 5.0)	4.0 (2.0, 4.0)
Comorbidities, *n* (%)									
Cardiovascular disease[c]	314 (5.0)	11 (8.7)	175 (4.9)	161 (5.4)	45 (5.0)	94 (5.1)	4 (4.7)	4 (23.5)	3 (13.0)
Stroke	88 (1.4)	4 (3.2)	65 (1.8)	61 (2.0)	7 (0.8)	16 (0.9)	3 (3.5)	1 (5.9)	0 (0)
PVD	119 (1.9)	2 (1.6)	55 (1.6)	47 (1.6)	24 (2.6)	40 (2.2)	0 (0)	1 (5.9)	1 (4.3)
MI	57 (0.9)	4 (3.2)	28 (0.8)	27 (0.9)	11 (1.2)	18 (1.0)	0 (0)	2 (11.8)	2 (8.7)
HF	119 (1.9)	2 (1.6)	63 (1.8)	60 (2.0)	20 (2.2)	36 (2.0)	1 (1.2)	1 (5.9)	0 (0)
Infection	598 (9.5)	10 (7.9)	377 (10.7)	319 (10.6)	63 (6.9)	158 (8.6)	8 (9.3)	1 (5.9)	1 (4.3)
Metastasis	753 (12.0)	17 (13.5)	449 (12.7)	387 (12.9)	93 (10.2)	211 (11.4)	11 (12.8)	2 (11.8)	4 (17.4)
Type 2 diabetes	944 (15.0)	24 (19.0)	509 (14.4)	463 (15.4)	127 (14.0)	308 (16.7)	14 (16.3)	5 (29.4)	5 (21.7)
CKD	206 (3.3)	8 (6.3)	94 (2.7)	84 (2.8)	33 (3.6)	79 (4.3)	4 (4.7)	3 (17.6)	1 (4.3)
Liver disease	75 (1.2)	1 (0.8)	30 (0.8)	25 (0.8)	15 (1.7)	30 (1.6)	1 (1.2)	0 (0)	0 (0)
COPD	109 (1.7)	5 (4.0)	24 (0.7)	20 (0.7)	27 (3.0)	58 (3.1)	2 (2.3)	3 (17.6)	0 (0)
HIV/AIDS	4 (0.1)	0 (0)	1 (0)	1 (0)	1 (0.1)	2 (0.1)	0 (0)	0 (0)	0 (0)
Organ transplantation	7 (0.1)	0 (0)	2 (0.1)	1 (0)	1 (0.1)	4 (0.2)	0 (0)	0 (0)	0 (0)
Neutropenia hospitalization	9 (0.1)	0 (0)	4 (0.1)	3 (0.1)	0 (0)	5 (0.3)	0 (0)	0 (0)	0 (0)

Characteristics	Any G-CSF		Any pegfilgrastim				Any sG-CSF		
	Any pegfilgrastim	Any sG-CSF	PFS	PFS ideal time (days 1–3)	OBI	Route unknown[a]	Filgrastim	Filgrastim-sndz	Tbo-filgrastim
Charlson comorbidity index, mean (SD)	5.0 (3.31)	5.4 (3.2)	5.0 (3.4)	5.1 (3.4)	4.8 (3.1)	5.1 (3.3)	5.6 (3.2)	5.2 (3.1)	4.7 (3.5)
Intermediate FN risk only		*N* = 4075			*N* = 3858			*N* = 217	
Number of patients	3858 (94.7)	217 (5.3)	2161 (56.0)	1885 (87.2)	474 (12.3)	1223 (31.7)	146 (67.3)	38 (17.5)	33 (15.2)
Mean age, years (SD)	67.5 (11.1)	67.2 (11.5)	66.6 (11.2)	67.2 (10.9)	67.9 (10.7)	69.0 (10.9)	66.7 (11.8)	69.7 (10.2)	66.4 (11.8)
Mean age category, years (SD)									
18–45 years	144 (3.7)	13 (6.0)	91 (4.2)	72 (3.8)	14 (3.0)	39 (3.2)	9 (6.2)	1 (2.6)	3 (9.1)
45–54 years	355 (9.2)	19 (8.8)	226 (10.5)	183 (9.7)	39 (8.2)	90 (7.4)	15 (10.3)	2 (5.3)	2 (6.1)
55–64 years	778 (20.2)	40 (18.4)	476 (22.0)	386 (20.5)	106 (22.4)	196 (16.0)	24 (16.4)	6 (15.8)	10 (30.3)
65–74 years	1526 (39.6)	82 (37.8)	833 (38.5)	751 (39.8)	180 (38.0)	513 (41.9)	57 (39.0)	19 (50.0)	6 (18.2)
≥75 years	1055 (27.3)	63 (29.0)	535 (24.8)	493 (26.2)	135 (28.5)	385 (31.5)	41 (28.1)	10 (26.3)	12 (36.4)
Sex, female	2271 (58.9)	148 (68.2)	1281 (59.3)	1123 (59.6)	281 (59.3)	709 (58.0)	98 (67.1)	28 (73.7)	22 (66.7)
Number of completed cycles, mean (SD)	3.8 (2.2)	3.3 (2.5)	4.0 (2.3)	4.1 (2.3)	3.9 (2.2)	3.6 (2.2)	3.0 (2.4)	4.2 (2.6)	3.2 (2.3)
Median (Q1, Q3)	4.0 (2.0, 6.0)	2.0 (1.0, 5.0)	4.0 (2.0, 6.0)	4.0 (2.0, 6.0)	4.0 (2.0, 6.0)	3.0 (1.0, 6.0)	2.0 (1.0, 5.0)	4.5 (1.0, 6.0)	2.0 (1.0, 5.0)
Comorbidities, *n* (%)									
Cardiovascular disease[c]	765 (19.8)	51 (23.5)	431 (19.9)	390 (20.7)	97 (20.5)	237 (19.4)	32 (21.9)	11 (28.9)	8 (24.2)
Stroke	207 (5.4)	7 (3.2)	138 (6.4)	130 (6.9)	19 (4.0)	50 (4.1)	2 (1.4)	3 (7.9)	2 (6.1)
PVD	326 (8.4)	34 (15.7)	180 (8.3)	164 (8.7)	39 (8.2)	107 (8.7)	24 (16.4)	5 (13.2)	5 (15.2)
MI	142 (3.7)	8 (3.7)	76 (3.5)	69 (3.7)	18 (3.8)	48 (3.9)	6 (4.1)	1 (2.6)	1 (3.0)
HF	275 (7.1)	14 (6.5)	144 (6.7)	130 (6.9)	44 (9.3)	87 (7.1)	8 (5.5)	4 (10.5)	2 (6.1)
Infection	1015 (26.3)	53 (24.4)	625 (28.9)	549 (29.1)	115 (24.3)	275 (22.5)	38 (26.0)	10 (26.3)	5 (15.2)
Metastasis	763 (19.8)	62 (28.6)	443 (20.5)	376 (19.9)	90 (19.0)	230 (18.8)	45 (30.8)	11 (28.9)	6 (18.2)
Type 2 diabetes	915 (23.7)	43 (19.8)	502 (23.2)	453 (24.0)	111 (23.4)	302 (24.7)	29 (19.9)	10 (26.3)	4 (12.1)
CKD	353 (9.1)	14 (6.5)	172 (8.0)	158 (8.4)	51 (10.8)	130 (10.6)	10 (6.8)	4 (10.5)	0 (0)
Liver disease	162 (4.2)	13 (6.0)	58 (2.7)	53 (2.8)	29 (6.1)	75 (6.1)	8 (5.5)	3 (7.9)	2 (6.1)
COPD	420 (10.9)	26 (12.0)	83 (3.8)	75 (4.0)	128 (27.0)	209 (17.1)	12 (8.2)	12 (31.6)	2 (6.1)
HIV/AIDS	10 (0.3)	0 (0)	2 (0.1)	1 (0.1)	4 (0.8)	4 (0.3)	0 (0)	0 (0)	0 (0)
Organ transplantation	11 (0.3)	1 (0.5)	6 (0.3)	4 (0.2)	3 (0.6)	2 (0.2)	1 (0.7)	0 (0)	0 (0)
Neutropenia hospitalization	51 (1.3)	1 (0.5)	32 (1.5)	26 (1.4)	4 (0.8)	15 (1.2)	1 (0.7)	0 (0)	0 (0)
Charlson comorbidity index, mean (SD)	6.8 (3.8)	7.2 (3.7)	7.0 (3.8)	7.0 (3.8)	6.5 (3.8)	6.6 (3.8)	7.3 (3.8)	7.4 (3.9)	6.8 (3.4)
Intermediate FN risk with ≥1 risk factors[b]		*N* = 2456			*N* = 2319			*N* = 137	
Number of patients	2319 (94.4)	137 (5.6)	1258 (54.2)	1114 (88.6)	305 (13.2)	756 (32.6)	92 (67.2)	29 (21.2)	16 (11.7)

Characteristics	Any G-CSF		Any pegfilgrastim				Any sG-CSF		
	Any pegfilgrastim	Any sG-CSF	PFS	PFS ideal time (days 1–3)	OBI	Route unknown[a]	Filgrastim	Filgrastim-sndz	Tbo-filgrastim
Mean age, years (SD)	69.5 (9.6)	68.9 (10.1)	68.7 (9.7)	69.2 (9.4)	70.1 (9.5)	70.4 (9.5)	69.1 (9.9)	69.6 (10.1)	66.3 (11.3)
Mean age category, years (SD)									
18–45 years	41 (1.8)	4 (2.9)	24 (1.9)	18 (1.6)	4 (1.3)	13 (1.7)	2 (2.2)	1 (3.4)	1 (6.3)
45–54 years	142 (6.1)	10 (7.3)	91 (7.2)	75 (6.7)	14 (4.6)	37 (4.9)	7 (7.6)	1 (3.4)	2 (12.5)
55–64 years	414 (17.9)	22 (16.1)	239 (19.0)	197 (17.7)	60 (19.7)	115 (15.2)	13 (14.1)	5 (17.2)	4 (25.0)
65–74 years	1002 43.2)	58 (42.3)	541 (43.0)	487 (43.7)	128 (42.0)	333 (44.0)	39 (42.4)	15 (51.7)	4 (25.0)
≥75 years	720 (31.0)	43 (31.4)	363 (28.9)	337 (30.3)	99 (32.5)	258 (34.1)	31 (33.7)	7 (24.1)	5 (31.3)
Sex, female	1320 (56.9)	86 (62.8)	709 (56.4)	628 (56.4)	185 (60.7)	426 (56.3)	57 (62.0)	20 (69.0)	9 (56.3)
Number of completed cycles, mean (SD)	3.7 (2.2)	3.3 (2.5)	3.8 (2.2)	3.9 (2.2)	3.7 (2.1)	3.6 (2.2)	2.9 (2.4)	4.4 (2.5)	3.5 (2.2)
Median (Q1, Q3)	4.0 (2.0, 6.0)	2.0 (1.0, 5.0)	4.0 (2.0, 6.0)	4.0 (2.0, 6.0)	4.0 (2.0, 6.0)	3.0 (2.0, 6.0)	2.0 (1.0, 5.0)	5.0 (2.0, 6.0)	3.5 (1.5, 5.0)
Comorbidities									
Cardiovascular disease[c]	765 (33.0)	51 (37.2)	431 (34.3)	390 (35.0)	97 (31.8)	237 (31.3)	32 (34.8)	11 (37.9)	8 (50.0)
Stroke	207 (8.9)	7 (5.1)	138 (11.0)	130 (11.7)	19 (6.2)	50 (6.6)	2 (2.2)	3 (10.3)	2 (12.5)
PVD	326 (14.1)	34 (24.8)	180 (14.3)	164 (14.7)	39 (12.8)	107 (14.2)	24 (26.1)	5 (17.2)	5 (31.3)
MI	142 (6.1)	8 (5.8)	76 (6.0)	69 (6.2)	18 (5.9)	48 (6.3)	6 (6.5)	1 (3.4)	1 (6.3)
HF	275 (11.9)	14 (10.2)	144 (11.4)	130 (11.7)	44 (14.4)	87 (11.5)	8 (8.7)	4 (13.8)	2 (12.5)
Infection	917 (39.5)	44 (32.1)	551 (43.8)	490 (44.0)	108 (35.4)	258 (34.1)	31 (33.7)	9 (31.0)	4 (25.0)
Metastasis	763 (32.9)	62 (45.3)	443 (35.2)	376 (33.8)	90 (29.5)	230 (30.4)	45 (48.9)	11 (37.9)	6 (37.5)
Type 2 diabetes	915 (39.5)	43 (31.4)	502 (39.9)	453 (40.7)	111 (36.4)	302 (39.9)	29 (31.5)	10 (34.5)	4 (25.0)
CKD	353 (15.2)	14 (10.2)	172 (13.7)	158 (14.2)	51 (16.7)	130 (17.2)	10 (10.9)	4 (13.8)	0 (0)
Liver disease	162 (7.0)	13 (9.5)	58 (4.6)	53 (4.8)	29 (9.5)	75 (9.9)	8 (8.7)	3 (10.3)	2 (12.5)
COPD	420 (18.1)	26 (19.0)	83 (6.6)	75 (6.7)	128 (42.0)	209 (27.6)	12 (13.0)	12 (41.4)	2 (12.5)
HIV/AIDS	9 (0.4)	0 (0)	2 (0.2)	1 (0.1)	3 (1.0)	4 (0.5)	0 (0)	0 (0)	0 (0)
Organ transplantation	11 (0.5)	1 (0.7)	6 (0.5)	4 (0.4)	3 (1.0)	2 (0.3)	1 (1.1)	0 (0)	0 (0)
Neutropenia hospitalization	48 (2.1)	1 (0.7)	29 (2.3)	26 (2.3)	4 (1.3)	15 (2.0)	1 (1.1)	0 (0)	0 (0)
Charlson comorbidity index, mean (SD)	8.0 (3.6)	8.4 (3.5)	8.2 (3.6)	8.1 (3.6)	7.5 (3.7)	7.8 (3.6)	8.7 (3.4)	8.6 (3.5)	6.4 (3.6)

Data presented as *n* (%) unless otherwise stated

Note: Column percentages are used except for row percentages for number of patients and FN risk (high, intermediate, or intermediate with ≥1 patient-level risk factors)

[a]Route unknown: pegfilgrastim users who could not be classified as pegfilgrastim PFS or OBI

[b]Risk factors include age >65 years, metastatic disease, diabetes mellitus, cardiovascular disease, COPD, liver or renal dysfunction, and HIV/AIDS

[c]Cardiovascular disease includes MI, HF, PVD, or stroke
CKD chronic kidney disease, *COPD* chronic obstructive pulmonary disease, *FN* febrile neutropenia, *HIV/AIDS* human immunodeficiency virus/acquired immune deficiency syndrome, *G-CSF* granulocyte colony-stimulating factor, *HF* heart failure, *MI* myocardial infarction, *OBI* on-body injector, *PFS* prefilled syringe, *prophylactic G-CSF* primary prophylaxis with granulocyte colony-stimulating factor, *PVD* peripheral vascular disease, *Q1* quartile 1, *Q3* quartile 3, *SD* standard deviation, *sG-CSF* short-acting granulocyte colony-stimulating factor

Online Resource 10 Proportion of patients completing all cycles in their chemotherapy course with the prophylactic pegfilgrastim (i.e., persistence), stratified by type of pegfilgrastim (March 31, 2015 to December 31, 2017)

	HR or IR	HR	IR	IR + ≥1 RF[a]
Pegfilgrastim PFS	*N* = 1040	*N* = 636	*N* = 404	*N* = 245
Proportion of cycles covered				
0–40%	190 (18.3)	123 (19.3)	67 (16.6)	48 (19.6)
41–60%	79 (7.6)	47 (7.4)	32 (7.9)	16 (6.5)
61–80%	148 (14.2)	126 (19.8)	22 (5.4)	12 (4.9)
81–100%	623 (59.9)	340 (53.5)	283 (70.0)	169 (69.0)
100%	612 (58.8)	332 (52.2)	280 (69.3)	168 (68.6)
Pegfilgrastim PFS ideal time (days 1–3)	*N* = 885	*N* = 539	*N* = 346	*N* = 207
Proportion of cycles covered				
0–40%	138 (15.6)	86 (16.0)	52 (15.0)	38 (18.4)
41–60%	64 (7.2)	35 (6.5)	29 (8.4)	15 (7.2)
61–80%	133 (15.0)	115 (21.3)	18 (5.2)	8 (3.9)
81–100%	550 (62.1)	303 (56.2)	247 (71.4)	146 (70.5)
100%	540 (61.0)	296 (54.9)	244 (70.5)	145 (70.0)
Pegfilgrastim OBI	*N* = 1383	*N* = 909	*N* = 474	*N* = 305
Proportion of cycles covered				
0–40%	200 (14.5)	124 (13.6)	76 (16.0)	48 (15.7)
41–60%	112 (8.1)	60 (6.6)	52 (11.0)	25 (8.2)
61–80%	186 (13.4)	165 (18.2)	21 (4.4)	16 (5.2)
81–100%	885 (64.0)	560 (61.6)	325 (68.6)	216 (70.8)
100%	871 (63.0)	551 (60.6)	320 (67.5)	212 (69.5)

Data are *n* (%)

[a]Risk factors include age >65 years, metastatic disease, diabetes mellitus, cardiovascular disease (myocardial infarction, heart failure, peripheral vascular disease, or stroke), COPD, liver or renal dysfunction, and HIV/AIDS

COPD chronic obstructive pulmonary disease, *FN* febrile neutropenia, *HIV/AIDS* human immunodeficiency virus/acquired immune deficiency syndrome, *HR* chemotherapy regimen with high risk for FN; *IR* chemotherapy regimen with intermediate risk for FN; *IR + ≥1 RF* chemotherapy regimen with intermediate risk for FN and ≥1 patient-level risk factor, *OBI* on-body injector, *PFS* prefilled syringe, *prophylactic pegfilgrastim* primary prophylaxis-pegfilgrastim, *RF* risk factor

Online Resource 11 Timing of PFS or pegfilgrastim-OBI for primary prophylaxis by FN risk category

	Cycle 1	Cycle 2	Cycle 3	Cycle 4	Cycle 5	Cycle 6	Cycle 7	Cycle 8	All cycles
Pegfilgrastim PFS									
High/intermediate FN risk	*N* = 5697	*N* = 4140	*N* = 3649	*N* = 3134	*N* = 1559	*N* = 1235	*N* = 341	*N* = 251	*N* = 20,006
<Day 0	12 (0.2)	9 (0.2)	3 (0.1)	4 (0.1)	2 (0.1)	2 (0.2)	1 (0.3)	0 (0)	33 (0.2)
Day 0	753 (13.2)	367 (8.9)	254 (7.0)	168 (5.4)	78 (5.0)	63 (5.1)	12 (3.5)	7 (2.8)	1702 (8.5)
Day 1	4084 (71.7)	3210 (77.5)	2896 (79.4)	2560 (81.7)	1226 (78.6)	967 (78.3)	242 (71.0)	175 (69.7)	15,360 (76.8)
Day 2	420 (7.4)	310 (7.5)	280 (7.7)	229 (7.3)	152 (9.7)	125 (10.1)	58 (17.0)	52 (20.7)	1626 (8.1)
Day 3	380 (6.7)	225 (5.4)	196 (5.4)	162 (5.2)	89 (5.7)	72 (5.8)	25 (7.3)	15 (6.0)	1164 (5.8)
Day 4	31 (0.5)	13 (0.3)	10 (0.3)	7 (0.2)	6 (0.4)	3 (0.2)	2 (0.6)	1 (0.4)	73 (0.4)
Day 5	17 (0.3)	6 (0.1)	10 (0.3)	4 (0.1)	6 (0.4)	3 (0.2)	1 (0.3)	1 (0.4)	48 (0.2)
High FN risk	*N* = 3536	*N* = 2649	*N* = 2401	*N* = 2136	*N* = 892	*N* = 707	*N* = 208	*N* = 155	*N* = 12,684
<Day 0	6 (0.2)	2 (0.1)	0 (0)	2 (0.1)	0 (0)	0 (0)	0 (0)	0 (0)	10 (0.1)
Day 0	507 (14.3)	253 (9.6)	175 (7.3)	116 (5.4)	44 (4.9)	35 (5)	5 (2.4)	3 (1.9)	1138 (9.0)
Day 1	2657 (75.1)	2145 (81.0)	1999 (83.3)	1822 (85.3)	755 (84.6)	608 (86.0)	185 (88.9)	140 (90.3)	10,311 (81.3)
Day 2	170 (4.8)	132 (5.0)	122 (5.1)	103 (4.8)	47 (5.3)	35 (5.0)	8 (3.8)	7 (4.5)	624 (4.9)
Day 3	172 (4.9)	109 (4.1)	99 (4.1)	87 (4.1)	42 (4.7)	29 (4.1)	10 (4.8)	5 (3.2)	553 (4.4)
Day 4	15 (0.4)	7 (0.3)	3 (0.1)	5 (0.2)	1 (0.1)	0 (0)	0 (0)	0 (0)	31 (0.2)
Day 5	9 (0.3)	1 (0)	3 (0.1)	1 (0)	3 (0.3)	0 (0)	0 (0)	0 (0)	17 (0.1)
Intermediate FN risk	*N* = 2161	*N* = 1491	*N* = 1248	*N* = 998	*N* = 667	*N* = 528	*N* = 133	*N* = 96	*N* = 7322
<Day 0	6 (0.3)	7 (0.5)	3 (0.2)	2 (0.2)	2 (0.3)	2 (0.4)	1 (0.8)	0 (0)	23 (0.3)
Day 0	246 (11.4)	114 (7.6)	79 (6.3)	52 (5.2)	34 (5.1)	28 (5.3)	7 (5.3)	4 (4.2)	564 (7.7)
Day 1	1427 (66.0)	1065 (71.4)	897 (71.9)	738 (73.9)	471 (70.6)	359 (68.0)	57 (42.9)	35 (36.5)	5049 (69.0)
Day 2	250 (11.6)	178 (11.9)	158 (12.7)	126 (12.6)	105 (15.7)	90 (17.0)	50 (37.6)	45 (46.9)	1002 (13.7)
Day 3	208 (9.6)	116 (7.8)	97 (7.8)	75 (7.5)	47 (7.0)	43 (8.1)	15 (11.3)	10 (10.4)	611 (8.3)
Day 4	16 (0.7)	6 (0.4)	7 (0.6)	2 (0.2)	5 (0.7)	3 (0.6)	2 (1.5)	1 (1.0)	42 (0.6)
Day 5	8 (0.4)	5 (0.3)	7 (0.6)	3 (0.3)	3 (0.4)	3 (0.6)	1 (0.8)	1 (1.0)	31 (0.4)
Intermediate FN risk with ≥1 risk factor[a]	*N* = 1258	*N* = 843	*N* = 701	*N* = 560	*N* = 376	*N* = 286	*N* = 81	*N* = 58	*N* = 4163

	Cycle 1	Cycle 2	Cycle 3	Cycle 4	Cycle 5	Cycle 6	Cycle 7	Cycle 8	All cycles
<Day 0	2 (0.2)	6 (0.7)	2 (0.3)	2 (0.4)	1 (0.3)	2 (0.7)	1 (1.2)	0 (0)	16 (0.4)
Day 0	128 (10.2)	56 (6.6)	44 (6.3)	31 (5.5)	21 (5.6)	17 (5.9)	5 (6.2)	4 (6.9)	306 (7.4)
Day 1	819 (65.1)	584 (69.3)	486 (69.3)	398 (71.1)	254 (67.6)	180 (62.9)	27 (33.3)	15 (25.9)	2763 (66.4)
Day 2	166 (13.2)	121 (14.4)	105 (15.0)	86 (15.4)	69 (18.4)	60 (21.0)	35 (43.2)	31 (53.4)	673 (16.2)
Day 3	129 (10.3)	69 (8.2)	58 (8.3)	40 (7.1)	27 (7.2)	23 (8.0)	11 (13.6)	8 (13.8)	365 (8.7)
Day 4	9 (0.7)	5 (0.6)	2 (0.3)	2 (0.4)	3 (0.8)	2 (0.7)	2 (2.5)	0 (0)	25 (0.6)
Day 5	5 (0.4)	2 (0.2)	4 (0.6)	1 (0.2)	1 (0.3)	2 (0.7)	0 (0)	0 (0)	15 (0.4)
Pegfilgrastim OBI									
High/intermediate FN risk	*N* = 1383	*N* = 1015	*N* = 906	*N* = 799	*N* = 366	*N* = 298	*N* = 58	*N* = 44	*N* = 4869
<Day 0	5 (0.4)	1 (0.1)	0 (0)	1 (0.1)	3 (0.8)	0 (0)	0 (0)	0 (0)	10 (0.2)
Day 0	1318 (95.3)	983 (96.8)	885 (97.7)	782 (97.9)	350 (95.6)	286 (96.0)	51 (87.9)	40 (90.9)	4695 (96.4)
Day 1	33 (2.4)	17 (1.7)	11 (1.2)	9 (1.1)	6 (1.6)	6 (2.0)	2 (3.4)	2 (4.5)	86 (1.8)
Day 2	18 (1.3)	12 (1.2)	8 (0.9)	6 (0.8)	6 (1.6)	5 (1.7)	4 (6.9)	1 (2.3)	60 (1.2)
Day 3	8 (0.6)	2 (0.2)	1 (0.1)	1 (0.1)	1 (0.3)	1 (0.3)	1 (1.7)	1 (2.3)	16 (0.3)
Day 5	1 (0.1)	0 (0)	1 (0.1)	0 (0)	0 (0)	0 (0)	0 (0)	0 (0)	2 (0)
High FN risk	*N* = 909	*N* = 706	*N* = 652	*N* = 599	*N* = 242	*N* = 198	*N* = 46	*N* = 37	*N* = 3389
<Day 0	2 (0.2)	0 (0)	0 (0)	1 (0.2)	3 (1.2)	0 (0)	0 (0)	0 (0)	6 (0.2)
Day 0	884 (97.2)	696 (98.6)	644 (98.8)	591 (98.7)	235 (97.1)	194 (98.0)	43 (93.5)	34 (91.9)	3321 (98.0)
Day 1	17 (1.9)	8 (1.1)	6 (0.9)	6 (1.0)	3 (1.2)	3 (1.5)	2 (4.3)	2 (5.4)	47 (1.4)
Day 3	5 (0.6)	2 (0.3)	1 (0.2)	1 (0.2)	1 (0.4)	1 (0.5)	1 (2.2)	1 (2.7)	13 (0.4)
Day 5	1 (0.1)	0 (0)	1 (0.2)	0 (0)	0 (0)	0 (0)	0 (0)	0 (0)	2 (0.1)
Intermediate FN risk	*N* = 474	*N* = 309	*N* = 254	*N* = 200	*N* = 124	*N* = 100	*N* = 12	*N* = 7	*N* = 1480
<Day 0	3 (0.6)	1 (0.3)	0 (0)	0 (0)	0 (0)	0 (0)	0 (0)	0 (0)	4 (0.3)
Day 0	434 (91.6)	287 (92.9)	241 (94.9)	191 (95.5)	115 (92.7)	92 (92.0)	8 (66.7)	6 (85.7)	1374 (92.8)
Day 1	16 (3.4)	9 (2.9)	5 (2.0)	3 (1.5)	3 (2.4)	3 (3.0)	0 (0)	0 (0)	39 (2.6)
Day 2	18 (3.8)	12 (3.9)	8 (3.1)	6 (3.0)	6 (4.8)	5 (5.0)	4 (33.3)	1 (14.3)	60 (4.1)
Day 3	3 (0.6)	0 (0)	0 (0)	0 (0)	0 (0)	0 (0)	0 (0)	0 (0)	3 (0.2)
Intermediate FN risk with ≥1 risk factor[a]	*N* = 305	*N* = 200	*N* = 158	*N* = 115	*N* = 71	*N* = 59	*N* = 8	*N* = 6	*N* = 922

	Cycle 1	Cycle 2	Cycle 3	Cycle 4	Cycle 5	Cycle 6	Cycle 7	Cycle 8	All cycles
<Day 0	3 (1.0)	1 (0.5)	0 (0)	0 (0)	0 (0)	0 (0)	0 (0)	0 (0)	4 (0.4)
Day 0	277 (90.8)	182 (91.0)	148 (93.7)	109 (94.8)	65 (91.5)	54 (91.5)	5 (62.5)	5 (83.3)	845 (91.6)
Day 1	8 (2.6)	5 (2.5)	2 (1.3)	1 (0.9)	1 (1.4)	1 (1.7)	0 (0)	0 (0)	18 (2.0)
Day 2	15 (4.9)	12 (6.0)	8 (5.1)	5 (4.3)	5 (7.0)	4 (6.8)	3 (37.5)	1 (16.7)	53 (5.7)
Day 3	2 (0.7)	0 (0)	0 (0)	0 (0)	0 (0)	0 (0)	0 (0)	0 (0)	2 (0.2)

Data are *n* (%)

[a]Risk factors include age >65 years, metastatic disease, diabetes mellitus, cardiovascular disease (myocardial infarction, heart failure, peripheral vascular disease, or stroke), COPD, liver or renal dysfunction, and HIV/AIDS

COPD chronic obstructive pulmonary disease, *FN* febrile neutropenia, *HIV/AIDS* human immunodeficiency virus/acquired immune deficiency syndrome, *OBI* on-body injector; *PFS* prefilled syringe
